# Functional analysis across model systems implicates ribosomal proteins in growth and proliferation defects associated with hypoplastic left heart syndrome

**DOI:** 10.7554/eLife.106231

**Published:** 2025-12-11

**Authors:** Tanja Nielsen, Anaïs Kervadec, Jeanne L Theis, Maria A Missinato, James Marchant, Michaela Romero, Katya Marchetti, Aashna Lamba, Xin-Xin I Zeng, Marie Berenguer, Stanley M Walls, Analyne Schroeder, Katja Birker, Greg Duester, Paul Grossfeld, Timothy J Nelson, Timothy M Olson, Karen Ocorr, Rolf Bodmer, Georg Vogler, Alexandre R Colas

**Affiliations:** 1 Center for Cardiovascular and Muscular Diseases, Sanford Burnham Prebys Medical Discovery Institute La Jolla United States; 2 Department of Biochemistry, Chemistry and Pharmacy, Freie Universität Berlin Berlin Germany; 3 Cardiovascular Genetics Research Laboratory, Mayo Clinic Rochester United States; 4 University of California San Diego, Rady Children’s Hospital San Diego United States; 5 Center for Regenerative Medicine, Division of Pediatric Cardiology, Department of Pediatric and Adolescent Medicine, Division of General Internal Medicine, Department of Molecular and Pharmacology and Experimental Therapeutics, Mayo Clinic Rochester United States; 6 Department of Cardiovascular Medicine, Division of Pediatric Cardiology, Department of Pediatric & Adolescent Medicine, Cardiovascular Genetics Research Laboratory, Mayo Clinic Rochester United States; GReD Laboratory/Clermont-Auvergne University Aubière France; Max Planck Institute for Heart and Lung Research Bad Nauheim Germany

**Keywords:** congenital heart disease, HLHS, ribosomal poteins, hiPSCs, zebrafish, *Drosophila*, Human

## Abstract

Hypoplastic left heart syndrome (HLHS) is the most lethal congenital heart disease (CHD) whose genetic basis remains elusive, likely due to oligogenic complexity. To identify regulators of cardiomyocyte (CM) proliferation relevant to HLHS, we performed a genome-wide siRNA screen in human iPSC-derived CMs, revealing ribosomal protein (RP) genes as the most prominent effectors of CM proliferation. Whole-genome sequencing of 25 HLHS proband–parent trios similarly showed enrichment of rare RP gene variants, including a damaging RPS15A promoter variant shared in a familial CHD case. Cross-species functional analyses demonstrated that perturbation of RP genes impairs cardiac growth: knockdown of RPS15A, RPS17, RPL26L1, RPL39, or RPS15 reduced CM proliferation, caused cardiac malformations in *Drosophila*, and produced hypoplastic or dysfunctional hearts in zebrafish. Genetic interactions between RP genes and key cardiac transcription factors (TBX5 and NKX2–7) further support their developmental role. Importantly, p53 suppression or Hippo activation partially rescued RP deficiency phenotypes. Together, these findings implicate RP genes as critical regulators of cardiogenesis and candidate contributors to HLHS.

## Introduction

Hypoplastic left heart syndrome (HLHS) accounts for 2–3% of all cases of congenital heart disease (CHD) Gordon et al., 2008; Reller et al., 2008 and is characterized by underdevelopment of the left ventricle, mitral and aortic valves, and aortic arch (Crucean et al., 2017). HLHS has a recognized genetic component based on its familial association with left-sided obstructive CHDs (Konno et al., 2010; Agopian et al., 2017; Martin et al., 2018). However, segregation analyses in multiplex HLHS-CHD families Martin et al., 2018 and genome-wide association studies of large cohorts Agopian et al., 2017 have lacked sufficient power to conclusively identify candidate HLHS-susceptibility genes with small to moderate effect sizes. Defects in a small number of genes involved in cardiogenesis, such as *NOTCH1* (Theis et al., 2015a), *NKX2–5* (Elliott et al., 2003), *MYH6* (Theis et al., 2015b), and *GATA4* Elliott et al., 2003; Zanon et al., 2017 have been implicated as contributors to HLHS, as well as multiple other CHDs. However, the variety of phenotypic manifestations of HLHS, together with numerous studies linking it to diverse genetic loci (Theis et al., 2015a; Elliott et al., 2003; Theis et al., 2015b; Dasgupta et al., 2001), suggests that HLHS is genetically heterogeneous and has a multigenic etiology (Yagi et al., 2018; Yaich et al., 1998).

It has also been hypothesized that the underdevelopment of the left ventricular (LV) myocardium includes restricted blood flow across the mitral valve and its hemodynamic effect during ventricular growth and development (‘no flow – no grow’; Grossfeld et al., 2009), as well as endocardial defects (Miao et al., 2020). Therefore, HLHS manifestation may be due to a combination of cardiomyocyte (CM) autonomous and non-autonomous genetic as well as mechanical effects, all of which likely affect proliferation and differentiation in the developing heart.

In a digenic HLHS mouse model, loss of both Sin3A Associated Protein 130 (*Sap130*) and the protocadherin *Pcdha9* causes decreased CM proliferation resulting in LV hypoplasia (Liu et al., 2017). In addition, LVs from HLHS patients have cardiac damage associated with CM proliferation defects compared to healthy subjects (Gaber et al., 2013). These studies suggest that intrinsically defective cardiac differentiation and impaired CM proliferation are likely contributing to HLHS-associated heart defects. Further strengthening this hypothesis, we found that HLHS-patient-derived hPSC-CMs exhibited reduced proliferation compared to the parents (Theis et al., 2020). There is a need to identify the potentially causative genes, which contribute to those processes described above and to elucidate their role in HLHS pathogenesis.

To advance CHD gene discovery, we have developed a multi-model systems platform enabling both to rigorously prioritize candidate genes from whole-genome sequencing (WGS) based on rare, predicted-damaging variants and their mode of inheritance, and to functionally characterize gene function in complementary and genetically tractable model systems: human-induced pluripotent stem cells (hPSCs), the fruit fly *Drosophila melanogaster*, and zebrafish *Danio rerio*. The genetic basis of cardiac development, originally uncovered in *Drosophila*, is fundamentally conserved across species (Bodmer, 1993; Bodmer, 1995; Cripps and Olson, 2002; Bodmer and Frasch, 2010) and ~80% of human disease genes have fly orthologs (Bier and Bodmer, 2004). In addition, the non-redundant fly genome, together with the simple structure and function of the fly heart, allows straightforward genotype–phenotype correlations. Zebrafish (Evans et al., 2010; Bakkers, 2011; Liu and Stainier, 2012), which has a two-chambered heart and can be easily manipulated using morpholino (MO) injections and CRISPR technologies to affect heart development and function. In addition, phenotypes can be directly observed in the developing larva for several days post-fertilization (Fink et al., 2009). Finally, CMs differentiated from hPSCs (hPSC-CMs) enable the identification and quantification of cellular phenotypes associated with human diseases, including HLHS (Theis et al., 2015a; Huang et al., 2011; Yu et al., 2018; Paige et al., 2020) and are amenable to large-scale functional screens (Diez-Cuñado et al., 2018; Murphy et al., 2021), thereby enabling them to serve both as a discovery and validation platform. This integrated approach allows for rapidly identifying novel HLHS-associated gene candidates and for characterizing their ability to regulate developmental processes associated with disease (e.g., hypoplasia) (Theis et al., 2020).

Defective CM proliferation is likely a hallmark of HLHS (Liu et al., 2017; Theis et al., 2020; Gaber et al., 2013), thus to uncover novel regulators of human CM proliferation with potential links to HLHS, we performed a whole-genome siRNA screen (18,055 genes) using an EdU assay in hPSC-CMs and identified ribosomal proteins (RPs) as the major class of genes controlling CM proliferation. In parallel, WGS of 25 poor-outcome HLHS proband–parent trios followed by unbiased variant filtering revealed an enrichment for rare, predicted-damaging variants in RP genes. Moreover, analysis of a high-value family (75H) comprised of an HLHS proband, his phenotypically normal parents, and a fifth-degree relative born with left-sided CHD led to the identification of a predicted-damaging variant in RP protein gene *RPS15A* that segregated with disease. Consistent with a central role for RPs in the regulation of heart development, heart-specific knockdown (KD) of HLHS-associated RPs in *Drosophila* caused severe phenotypes, including near-complete heart loss and lethality. Similarly, in zebrafish, *rps15a* CRISPR mutants and morphants had greatly reduced heart size and CM number, looping defects, and diminished contractility. In this context, a multi-model system evaluation (hPSC-CMs, flies, and zebrafish) of RP function shows their important role in heart growth and differentiation and CM proliferation by modulating the p53, Myc, and Hippo pathways. Moreover, we find evidence that RPs may regulate heart development and CM proliferation in a cardiac-specific manner by synergistically interacting with core cardiac transcription factors (cTFs) such as T-box, Nkx, and Gata factors. In sum, here we show that RP genes are potent regulators of CM proliferation and cardiac growth and differentiation, consistent with a potentially critical role in the hypoplastic phenotype of HLHS. We suggest that RP genes are an emerging class of novel genetic effectors in HLHS.

## Results

### Whole-genome siRNA screen identifies RPs as central regulators of CM proliferation

**Figure 1.**
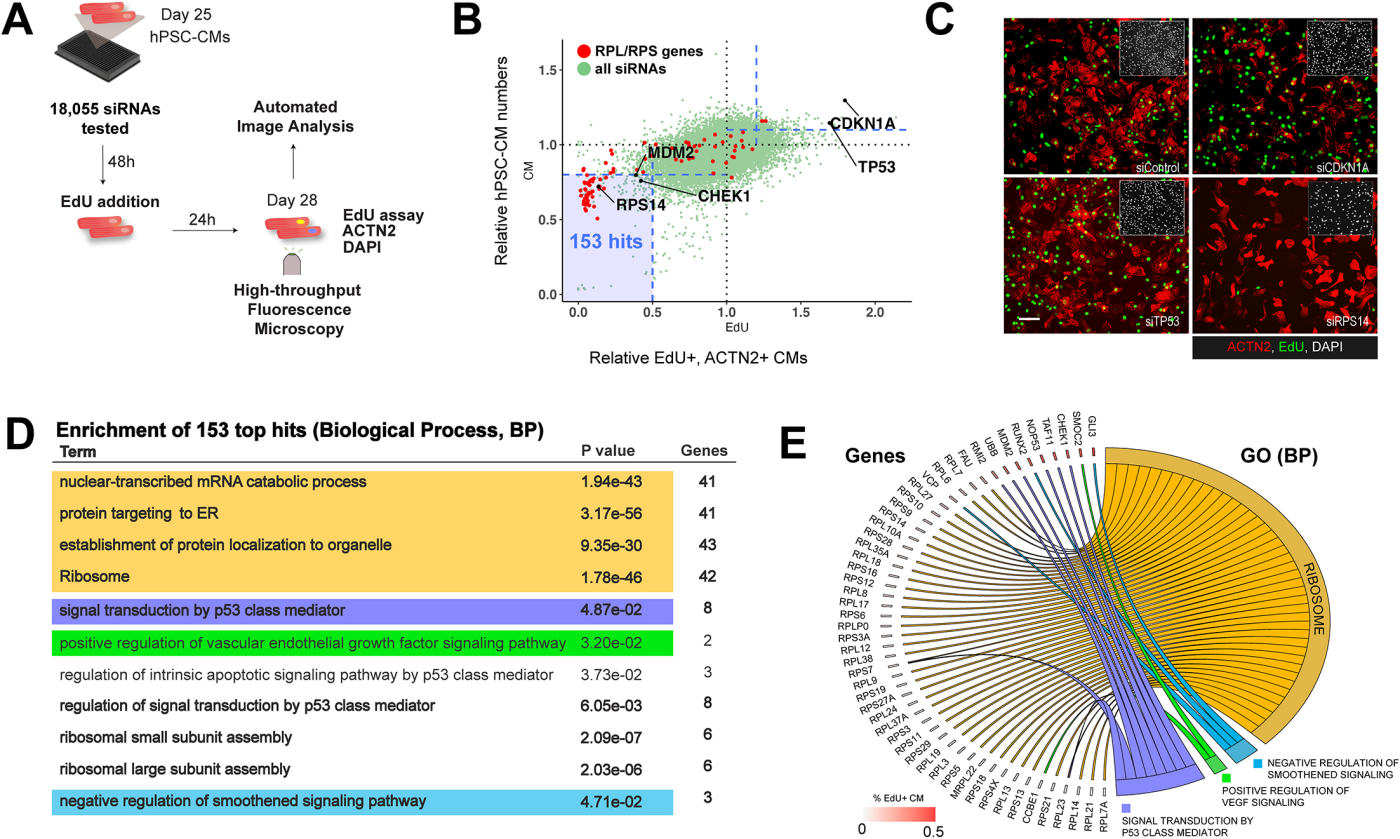
Whole-genome siRNA screen identified ribosomal proteins as agonists of cardiomyocyte (CM) proliferation. (**A**) High-throughput iPSC-derived CM proliferation screen overview. (**B**) Screen result showing normalized % EdU+ CMs (*X*-axis) and relative total number of CMs (*Y*-axis) upon knockdown of genome-wide siRNAs (18,055 siRNAs). siRNAs for RPL and RPS genes highlighted in red. (**C**) Representative immunofluorescence images of proliferation (EdU incorporation, green, CM marker ACTN2, red) of induced hPSC-CMs upon *TP53* and *RPS14* knockdown. Insets: nuclei (DAPI). (**D**) Gene ontology enrichment analysis for whole-genome sequencing (WGS) hits (BP, biological process; FDR-corrected analysis using gprofiler2). (**E**) Overview of hits corresponding to top 4 non-redundant BP categories.

Based on previous and our recent data (Liu et al., 2017; Gaber et al., 2013; Theis et al., 2020), impaired CM proliferation is emerging as an important mechanism in HLHS pathogenesis. Thus, to comprehensively map the human genome for novel regulators of CM proliferation, we screened a library of 18,055 siRNAs for their ability to modulate proliferation in human iPSC-derived CMs (hPSC-CMs). To assess CM proliferation, we used a dual read-out and quantified both DNA synthesis using EdU incorporation (Diez-Cuñado et al., 2018) and total number of CMs, 3 days after treatment (Figure 1A–C). Briefly, siRNAs were transfected into 25-day-old hPSC-CMs (Yu et al., 2018; Cunningham et al., 2017). After 2 days, EdU was added to the wells for 24 hr. On day 3, cells were fixed and co-stained for sarcomeric protein alpha-Actinin (ACTN2), EdU, and DAPI. Next, the number of EdU/alpha-Actinin double-positive cells and the total number of CMs were determined using a commercially available high-throughput image analysis software (MetaXpress, Molecular Devices) (Diez-Cuñado et al., 2018). Note that alpha-Actinin-, EdU+ cells which typically express fibroblast markers (i.e POSTN) (Cunningham et al., 2017), were excluded from this analysis, to focus on CM biology only. In total, we found 153 siRNAs that decreased proliferation (<0.5-fold EdU incorporation) and number of CMs (<0.8-fold), and 162 siRNAs that increased EdU (>1.2-fold) and CM number (>1.1-fold; Figure 1B, C and Supplementary file 1). Consistent with previous studies (Zhang et al., 2003; Yuan et al., 2014), KD of cell cycle checkpoint agonist, CHEK1 and MDM2, the negative regulator of TP53, both were among top hits that reduced CM proliferation. As expected, we identified TP53 and CDKN1A among the genes that significantly increased CM cell number upon KD (Figure 1B, C). Next, to gain insight into the molecular pathways regulating proliferation in hPSC-CMs, we performed gene ontology (GO) term analysis of hits decreasing EdU incorporation or CM numbers and found an enrichment for genes associated with ‘translation/ribosome’ and p53 signaling as top hits (Figure 1D, E). Consistent with these observations, we had previously found that the p53 pathway is dysregulated in hPSCs differentiated from an HLHS family trio (Theis et al., 2020). Remarkably, the most represented gene family is RPs, KD of which caused consistently the strongest inhibition of cell proliferation (Figure 1B). Next, since the primary screen was performed as a single data point, we next sought to validate these findings by re-testing all 80 RP genes for function (Zhou et al., 2015) using a distinct set of siRNAs in biological quadruplicate conditions. Remarkably and consistent with the primary screen, KD of 59/80 RPs significantly reduced proliferation as compared to siControl (p < 0.05; Figure 1—figure supplement 1A, B). In this context, further functional testing in fibroblasts revealed that the antiproliferative effect of RP loss of function is cell type independent (Figure 1—figure supplement 1C, D). Together, these findings establish RP function as essential for cell proliferation, including in hPSC-CMs, and suggest that RPs may represent a previously unrecognized class of regulators of ventricular growth during embryonic development.

**Figure 1—figure supplement 1.**
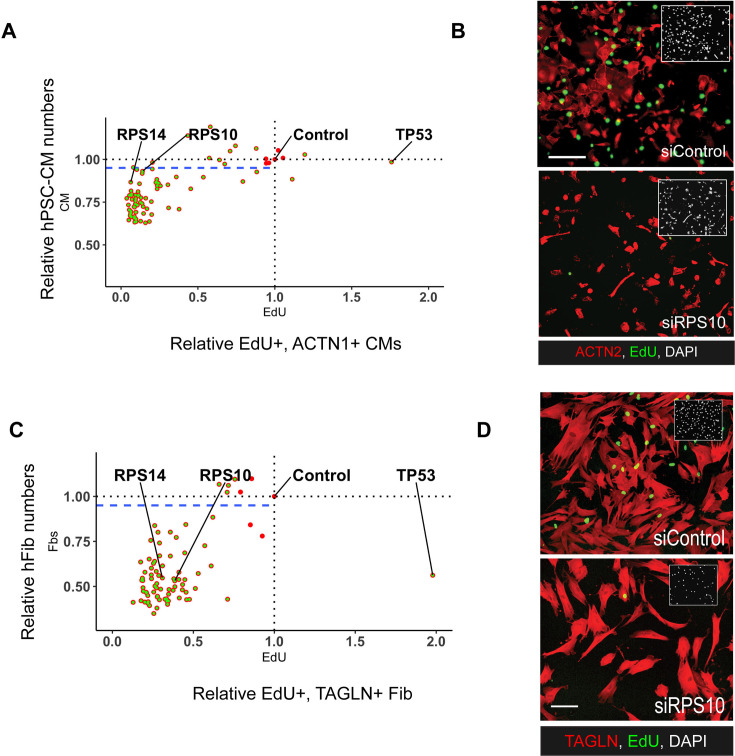
Functional validation of RP–mediated control of proliferation in hiPSC-CMs and human dermal fibroblasts. (**A, B**) Confirmatory siRNA screening for all RP genes confirms critical role for RPs in cardiomyocyte (CM) proliferation. (**C, D**) KD of RPs reduces proliferation in human fibroblasts.

### Enrichment of RP gene variants in an HLHS patient cohort with poor clinical outcome

**Figure 2.**
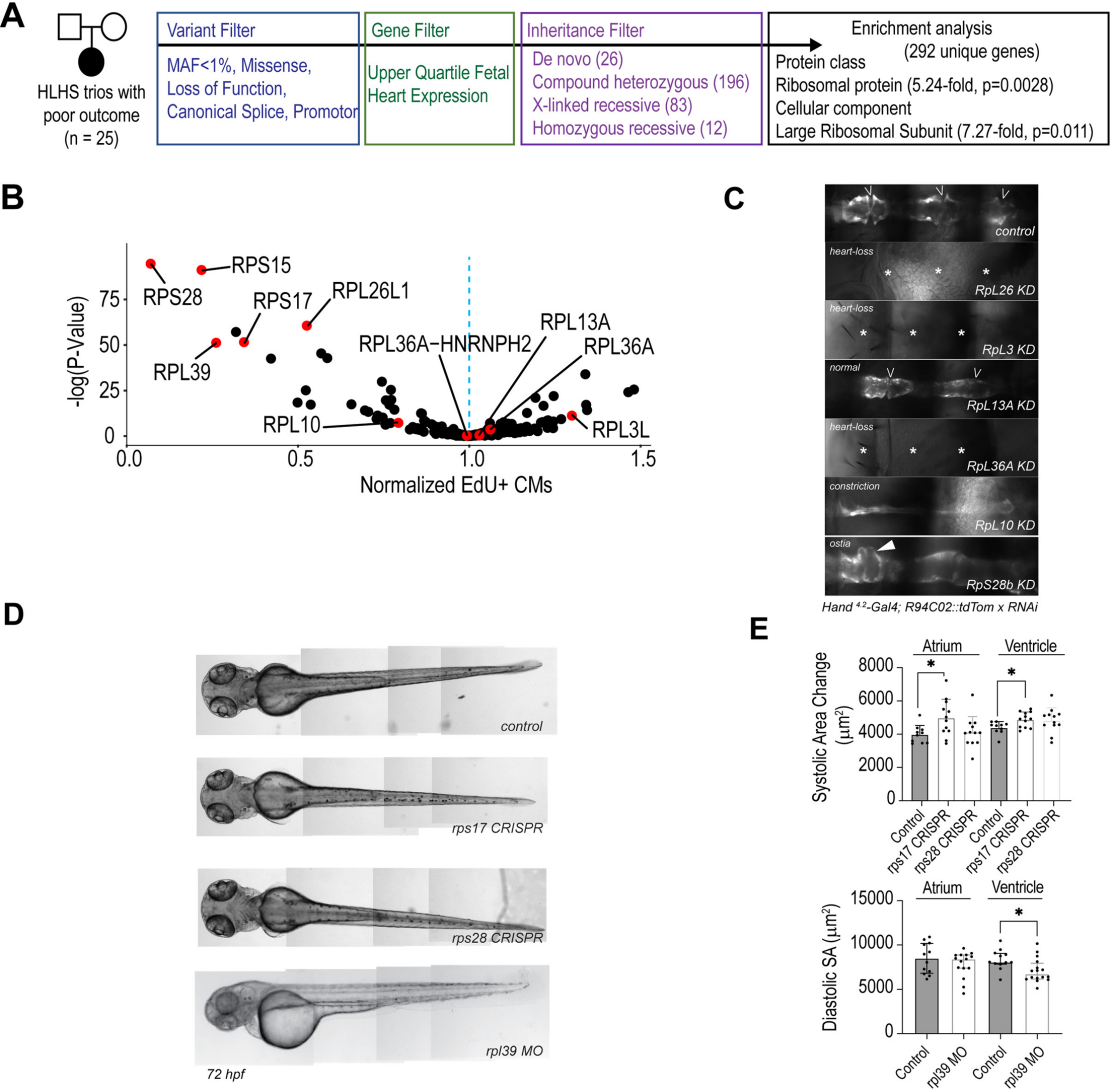
Ribosomal gene variants identified in hypoplastic left heart syndrome (HLHS). (**A**) Gene prioritization scheme of 25 poor-outcome proband–parent trios. (**B**) Testing 292 HLHS candidate genes from all poor-outcome families in cardiomyocytes (CMs) identified RPs as major regulators of hPSC-CM proliferation (normalized fraction of ACTN2+/EdU+ cells). (**C**) *Drosophila* cardiac phenotypes induced by loss of RP genes affected in HLHS patients with poor outcome. The heart is visualized by RFP expression specifically in CMs (R94C02::tdTom). Knockdown is achieved by sustained Gal4/UAS activity using Hand4.2-Gal4. (**D**) Wild-type zebrafish larva, *rps17* and *rps28* CRISPR mutants, and *rpl39* morphants at 72 hpf. *rpl39* morphants, injected with 1 ng MO in lateral view, show mild edema. (**E**) Systolic surface area (SA) upon *rps17* and *rps28* CRISPR, and diastolic SA after *rpl39* MO in the atrium and ventricle of zebrafish hearts.

To identify candidate genes involved in HLHS pathogenesis, we performed WGS, variant filtering, and enrichment analysis (see also, Theis et al., 2020), and Methods: WGS and bioinformatic strategies in a cohort of 25 HLHS patient–parent trios with poor clinical outcome defined as either prenatal – restrictive atrial septal defect (*n* = 2); postnatal – reduced right ventricular ejection fraction following stage II or III surgical palliation (*n* = 19); protein-losing enteropathy (*n* = 2); or cardiac transplantation/failed surgical palliation (*n* = 2). The 25 probands were comprised of 18 males and 7 females, 24 of whom were of white ancestry. The HLHS phenotype with respect to valve morphology (mitral atresia, MA; mitral stenosis, MS; aortic atresia, AA; aortic stenosis, AS) was MA/AA in 10, MS/AS in 9, MA/AA in 4, MA/AS in 1, and unknown in 1. CHD in the parents was excluded by echocardiography in all but two families, in which a bicuspid aortic valve was present in the mother. Genomic sequences were filtered for uncommon (minor allele frequency [MAF] <1%) de novo and recessive variants in coding and regulatory regions of genes expressed in the developing heart and predicted to alter protein structure or expression, yielding 292 unique HLHS candidate genes that primarily fit a recessive mode of inheritance (Figure 2A and Supplementary file 2). To determine whether certain gene networks were over-represented among these variants, we used two online bioinformatics tools (STRING and PANTHER; Mi et al., 2019; Szklarczyk et al., 2019). After applying Fisher’s exact test and false discovery rate corrections, RPs were the most enriched class of proteins when compared to the reference proteome, which includes data annotated by protein class (5.24-fold, p = 0.0028), and cellular component (7.27-fold, p = 0.011). In total, 14 variants found in 9 RP genes among 6 HLHS probands were identified, most fitting a recessive inheritance disease model (Table 1). These RP variants encompass mutations in upstream promoter regions that potentially affect transcription factor-binding sites, as well as non-synonymous substitutions inside the protein coding regions.

**Table 1.** Variants in ribosomal genes in hypoplastic left heart syndrome (HLHS) probands.

Gene	Proband (age, sex)	Mode of inheritance	Variant	Type	MAF%	CADD score	TFBS affected	hPSC-CM proliferation	Fly gene and defects	Zebrafish gene and defects	Patient outcome
RPL26L1	145H (3y, m)	Compound heterozygous	–1248A>G; V97M	Regulatory; missense	0.029; 0.032	-; 21.3	Pdx1; NFE2L1::MAFG; FOXC1	Reduced	RpL26: lethal, no heart	rpl26: n.t.	Restrictive ASD, PLE
RPL36A		X-linked recessive	–1321C>T	Regulatory	0.055	-	PAX2	No effect	RpL36A: lethal, no heart	rpl36a: n.t.	
RPS15		Compound heterozygous	–1558C>T; T101S	Regulatory; missense	0; 0.102	-; 23.5	FOXC1	Reduced	RpS15: lethal	rps15: n.t.	
RPL39	151H (20y, m)	X-linked recessive	–1359T>C	Regulatory	0.653	-	HOXA5	Reduced	RpL39: lethal	rpl39: morphants mild edema, reduced ventricular size	Reduced RV function
RPL3L	96H (22m, m)	Compound heterozygous	R200Q; R242W	Missense; missense	0.966; 0.432	21.2; 14.94	-	Elevated	RpL3: no heart	rpl3: n.t.	Reduced RV function
RPL13A	201H	Compound heterozygous	–92–645C>T; –29–191C>T	Regulatory; regulatory	0.72; 0.046	-	FOXD1; GATA2; ETS1; ELF5; FOXC1	No effect	RpL13A: no phenotype	rpl13a: n.t.	Failing Fontan circulation, transplant at 14y
RPS17	325H	Homozygous recessive	S136N	Missense	0	<10	-	Reduced	RpS17: lethal	rps17: CRISPR mutants show systolic atrial dysfunction, shortened heart period	Reduced RVEF and increased RVEDP at 9m
RPL10	76H	X-linked recessive	24–218G>A	Regulatory	0.583	-	ELK1; ETS1; SPIB; POLR2A; HEY1; Hltf	Reduced	RpL10: constricted	rpl10: n.t.	Reduced RVEF at 9y
RPS28		Compound heterozygous	–589G>A; –505A>T	Regulatory; regulatory	0.061; 0.08	-	CTCF	Reduced	RpS28b: ostia defect	rps28: CRISPR mutants show no heart phenotype	

MAF – minor allele frequency; TFBS – transcription factor-binding site; n.t. – not tested; ASD – atrial septal defect; PLE – protein-losing enteropathy; RV – right ventricle; RVEF – right ventricular ejection fraction; RVEDP – right ventricular end diastolic pressure.

Next, all 292 prioritized candidate genes were systematically evaluated for effects on the proliferation of hPSC-CMs in vitro and for cardiac differentiation of fly hearts in vivo. Again, loss of RP function (*RPL39*, *RPL26L1*, *RPS15*, *RPS17*, *RSPS28*) caused the most severe phenotypes on proliferation in hPSC-CMs (Figure 2B, Supplementary file 3), which was consistent with our genome-wide proliferation screen (see Figure 1B). In the fly, KD of RPs in the cardiac lineage and throughout embryonic development was achieved using a *Hand^4.2^*-Gal4 driver (see Brand and Perrimon, 1993 and methods) and led to partial or complete heart loss (*RpL26*, *RpL36A*, *RpL3*) or caused lethality (*RpS17*, *RpL39*, *RpS15*, *RpL26*, *RpL36A*) (Figure 2C). Also, KD of *RpL10* gave rise to severely constricted hearts, while KD of *RpS28b* caused inflow tract defects (Figure 2C). Finally, functional testing of RPs in zebrafish embryos revealed that KD of *rpl39* leads to reduced ventricle size, whereas *rps17* CRISPR mutants exhibit systolic dysfunction (Figure 2D, E). In sum, our patient-centric approach (cohort of 25 probands) identifies RPs as the most enriched gene category affected by HLHS-associated variants (STRING, PANTHER analysis). In this context, systematic testing in multi-model systems (fly, zebrafish, hPSC-CMs) identifies RPs as most potent regulators of cardiac differentiation, proliferation, and contractility among HLHS-associated genes. Collectively, these findings highlight that RPs regulate critical steps of cardiogenesis that are also found to be defective in HLHS probands (Gaber et al., 2013; Liu et al., 2017) and thus suggest a potential link between RP biology and HLHS phenotypic etiology.

### RPS15A variant associated with HLHS in familial CHD

To further establish a phenotypic link between RP function and HLHS-associated phenotypes, we selected a rare familial CHD case (75H) (Figure 3—figure supplement 1A), that is comprised of a young teenager with HLHS (MS/AS) and normal ventricular function following Fontan operation, his phenotypically normal parents, and a fifth-degree female relative born with left-sided CHD (bicuspid aortic valve and coarctation of the aorta). Based on these observations, we hypothesized the presence of a heterozygous driver variant exhibiting incomplete penetrance and variable expression. To investigate further, we filtered WGS data for rare, predicted-damaging variants in coding and regulatory regions. This analysis identified six prioritized variants (Supplementary file 4), including one located in the *RPS15A* gene locus, shared by the HLHS proband, the unaffected mother, and the proband’s fifth-degree relative with CHD.

**Figure 3.**
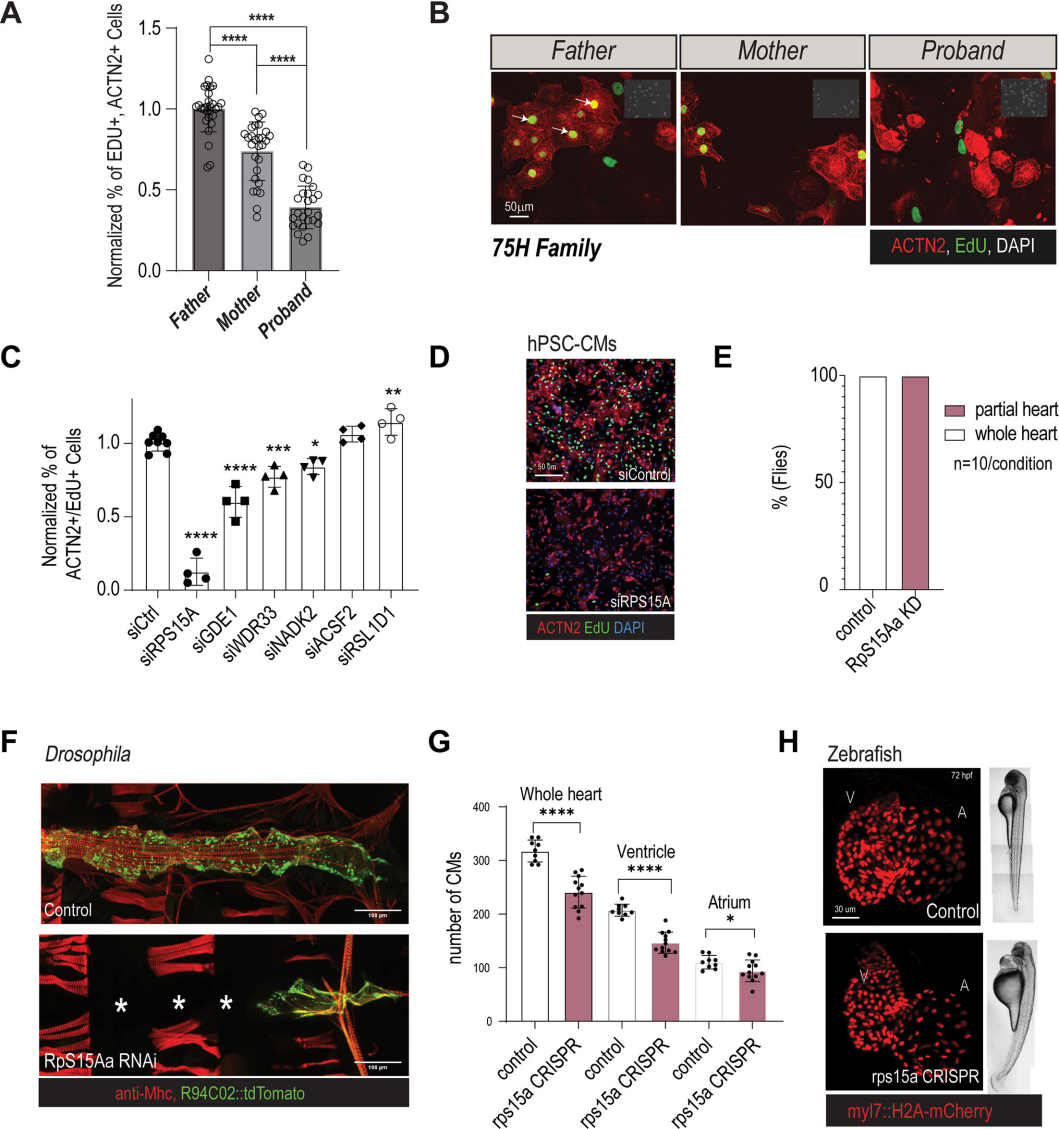
Characterization of RPS15A from the 75H hypoplastic left heart syndrome (HLHS) index family. (**A**–**C**) Prioritized candidate genes from the 75H family and relative hPSC-cardiomyocyte (CM) proliferation capacity upon KD. (**D**) Representative immunofluorescence images of proliferation (EdU incorporation, green; CM marker ACTN2, red) of induced hPSC-CMs upon siRPS15A knockdown. (**E**) Heart-specific KD of *RpS15Aa* in *Drosophila* adult hearts causes loss of heart tissue and is fully penetrant. (**F**) Representative immunofluorescence images of control and *RpS15Aa*-RNAi show partial heart loss (myosin heavy chain, Mhc, red; heart tissue-reporter, green). (**G**) CRISPR-mediated loss of *rps15a* in F_0_ larval zebrafish hearts causes decrease in CM number. (**H**) Representative immunofluorescence of hearts and whole-mount images of control and *rps15a*-CRISPR F_0_ larval hearts (CM nuclei reporter, red).

We next investigated whether family members carrying these variants (the mother and proband) exhibit defects in CM proliferation compared to the father. Notably, analysis of EdU incorporation in CMs from the parent–proband trio revealed significantly reduced proliferation in both the proband and mother compared to the father. However, the proband displayed a markedly more severe phenotype than the mother (Figure 3A, B). Interestingly, this reduction in proliferation appeared to be specific to CMs, as EdU incorporation in the non-CM cell population showed no significant differences among the family members (Figure 3—figure supplement 1B). Collectively, these findings suggest a phenotypic association between the presence of the variants and impaired CM proliferation.

**Figure 3—figure supplement 1.**
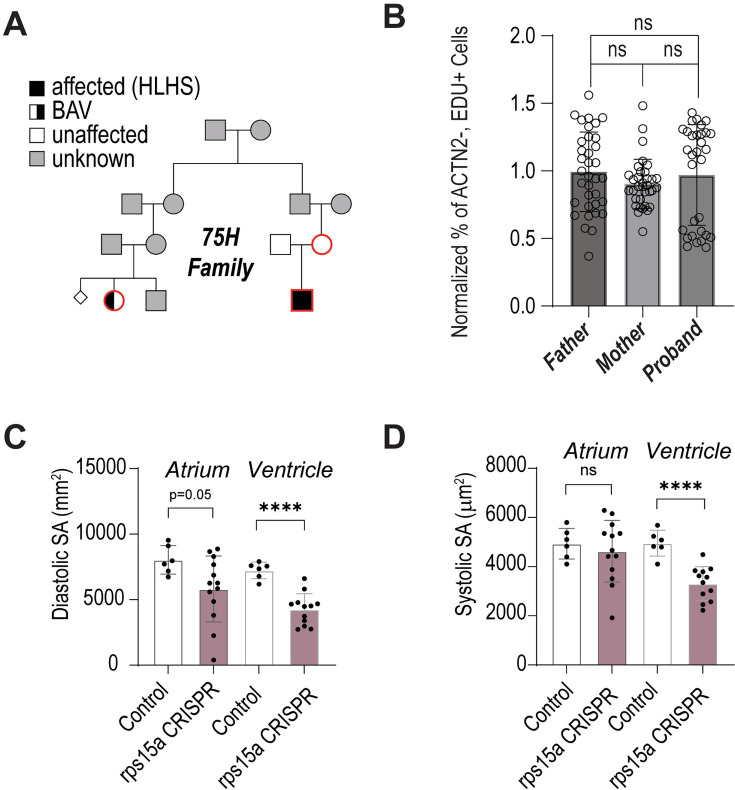
Model organism phenotypes caused by loss of RP genes. (**A**) Pedigree of the extended 75H family investigated for shared variants between hypoplastic left heart syndrome (HLHS) proband (black square) and distant cousin with congenital heart disease (CHD) (half-filled circle). (**B**, **C**) Diastolic and (**D**) systolic surface area after rps15a CRISPR. Statistics: Mann–Whitney test, *p < 0.05, **p < 0.01, ***p < 0.001, ****p < 0.0001.

**Figure 3—figure supplement 2.**
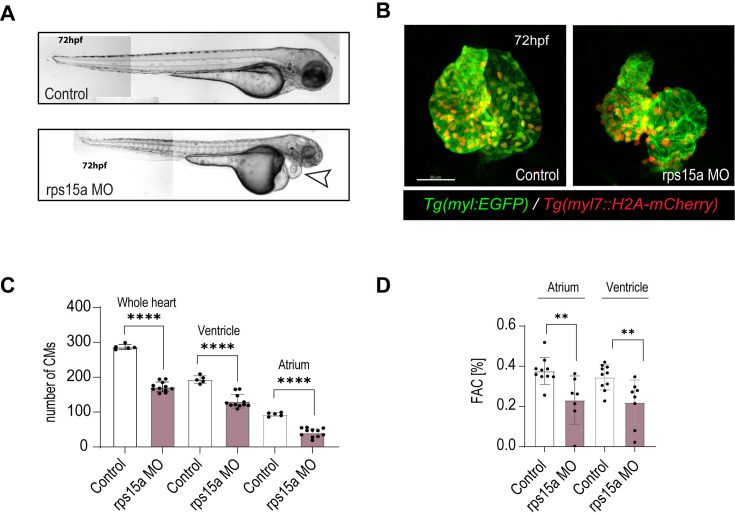
*rps15a* zebrafish larval morphants show heart dysfunction and reduction in cardiomyocyte (CM) number. (**A**) Lateral view (head to the right) of a wild-type control zebrafish larva (top) and a morphant (bottom) following injection of 2 ng/µl *rps15a* morpholino (MO). The morphant exhibits near-normal body/tail but notable pericardial edema (arrowhead). (**B**) Control (left) and morphant hearts (right) stained with Tg(*myl7:EGFP*) and Tg(*myl7:H2A-mCherry*). Note the smaller heart with aberrant looping in morphant heart. (**C**) Total CM counts (mCherry+ cells) in control and *rps15a* morphants shown in (**B**). (**D**) Fractional area change (FAC) in control and *rps15a* morphants. Student’s *t*-test, *p < 0.05, **p < 0.01, ***p < 0.001, ****p < 0.0001.

**Figure 3—figure supplement 3.**
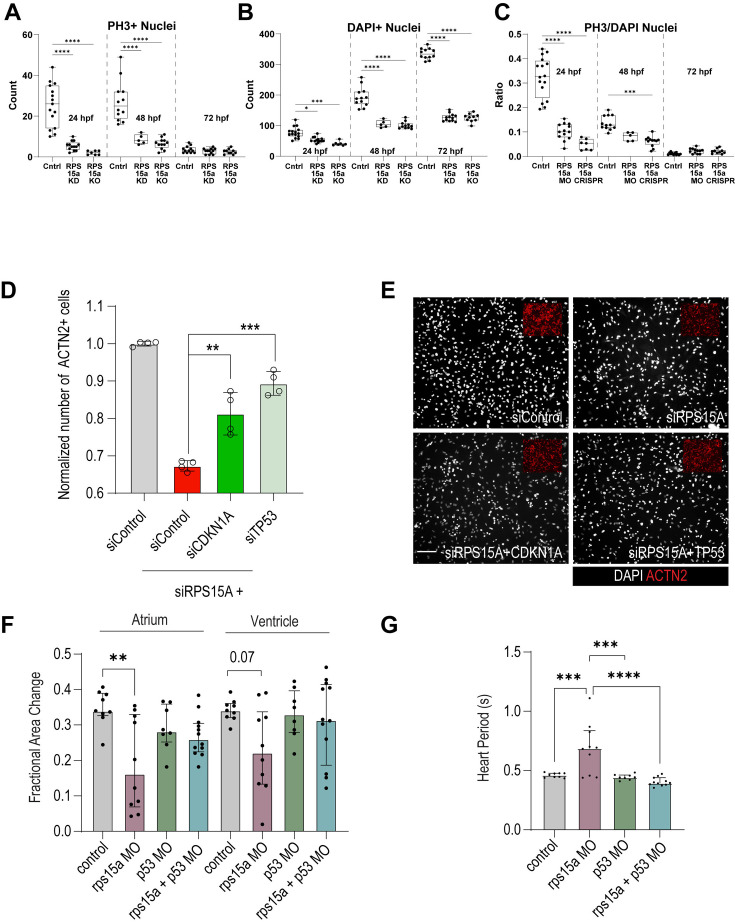
Proliferation and heart function depend on RPS15A and are regulated via TP53 in cardiomyocytes (CMs) and zebrafish. Cardiac proliferation (by PH3, **A**), total CM number (DAPI, **B**) and ratio of proliferating CMs (**C**) assessed for *rps15a* morphants (KD) and CRISPR (KO) for three timepoints, 24/48/72 hpf (**D, E**) The number of *siRPS15A-*treated CMs is strongly reduced, which can be attenuated by co-KD of *CDKN1A* or *TP53*. (**F, G**) Loss of *rps15a* causes strong reduction in zebrafish heart contractility (measured by fractional area change, **C**) and heart period (**D**), which can be rescued by co-KD of *p53* in both zebrafish heart atrium and ventricle.

Finally, to determine the potential relative contribution of the six genes harboring rare and damaging variants in the regulation of CM proliferation, we performed functional KD experiments in hPSC-CMs in vitro. Remarkably, while KD of 5 of the other prioritized genes from the 75H family also resulted in moderate reduced CM proliferation, RPS15A KD produced the most pronounced effect, with over an 85% reduction in proliferation, emerging as the top candidate (Figure 3C, D). Together, these findings indicate that RPS15A plays a critical role in regulating CM proliferation in human pluripotent stem cells (hPSCs) and may influence heart developmental processes that are disrupted in HLHS.

### RPS15A KD in *Drosophila* and zebrafish causes severe cardiac proliferation and differentiation defects

To characterize the role of *RPS15A* during heart development, we first induced a heart-specific KD of *RpS15Aa* in flies (see methods), which caused a partial or complete loss of the heart (Figure 3E, F). Note that this phenotype is consistent with our results above and a previous study involving another RP gene (*RPL13*) (Schroeder et al., 2019).

Next, to evaluate the role of *RP* genes during heart development, we knocked out *rps15a* in zebrafish (*D. rerio*) using CRISPR (Evans et al., 2010; Bakkers, 2011; Liu and Stainier, 2012). We examined F_0_ larva at 72 hpf (hours post fertilization) using high-speed digital imaging (SOHA; Fink et al., 2009, see also methods) and confocal microscopy to monitor heart size and CM numbers, respectively. Remarkably, *rps15a* mutant hearts were smaller in size (Figure 3—figure supplement 1C, D), with reduced CM numbers (Figure 3G, H). We observed similar effects in response to injection of a *rps15a* MO compared to uninjected larva (Uechi et al., 2006; Ikeda et al., 2017; Figure 3—figure supplement 2A–D). In this context, we also noted that the overall body plan and morphology of *rps15a* CRISPR mutants or morphants were largely unaffected, except for a mild pericardial edema, a possible indicator of heart dysfunction (Figure 3H; Miura and Yelon, 2011).

We quantified CM number using nuclear markers in Tg(*myl7:eGFP*); Tg(*myl7:H2A-mCherry*) embryos 72 hpf. CM numbers were predominantly reduced in the ventricle compared to the atrium (Figure 3G, H). We also assessed total cardiac cell numbers and cardiac cell proliferation by phospho-histone H3 (PH3) immunostaining and DAPI to quantify total cardiac cells. We again found that the total cell numbers were significantly reduced in both rps15a morphants and crispants (Figure 3—figure supplement 3A–C). Importantly, the proportion of proliferating (PH3+) cardiac cells was also decreased in rps15a morphants and crispants at both 24 and 48 hpf, but not at 72 hpf when proliferation normally declines (Figure 3—figure supplement 3A–C). Collectively, these data support the hypothesis that *RPS15A* is required for CM proliferation and heart morphogenesis (hPSC-CMs, fly, and zebrafish), thus suggesting a potential role for RPs as genetic drivers of hypoplasticity observed in HLHS.

### RPs control CM cell cycle by regulating TP53 pathway activity

**Figure 4.**
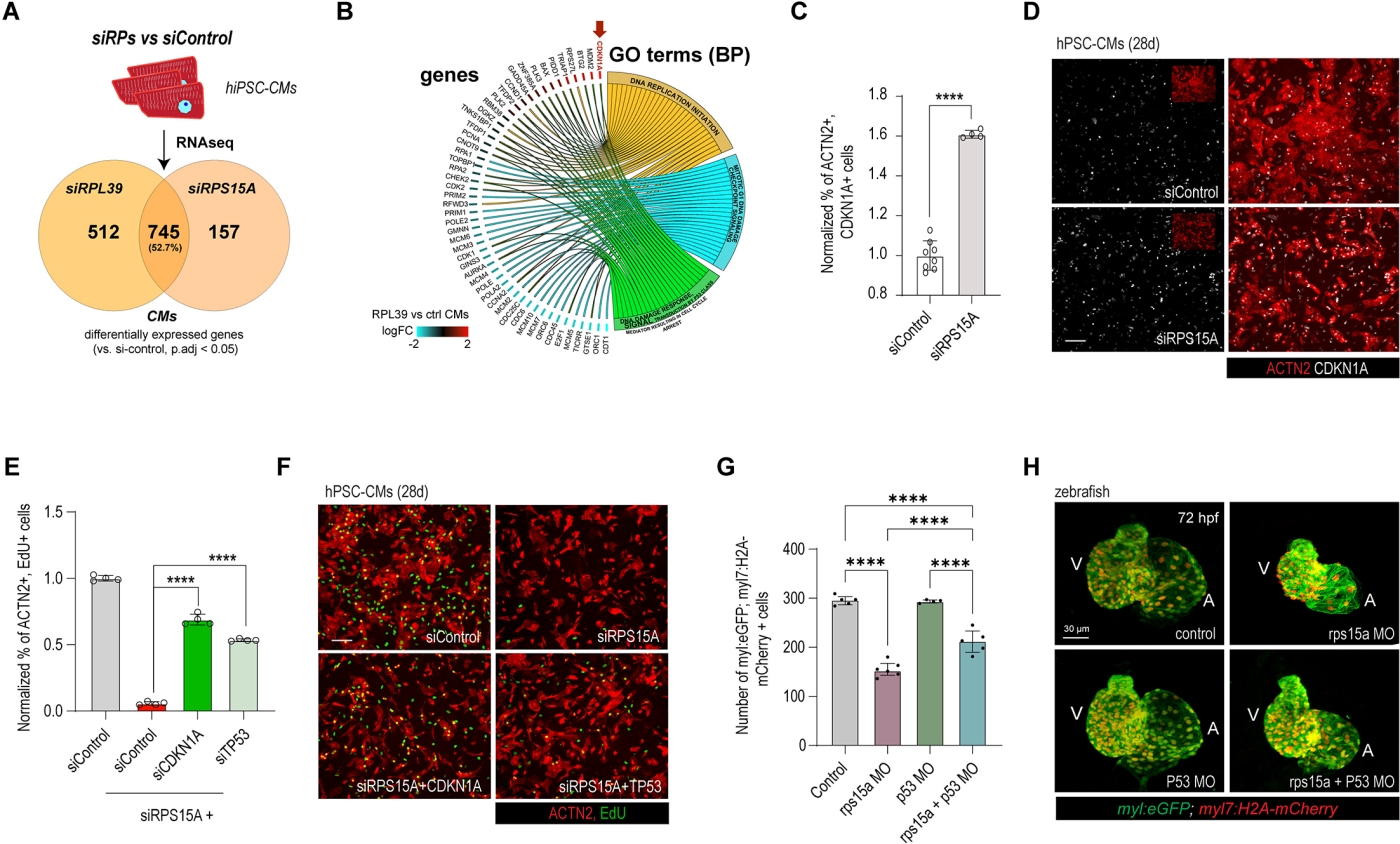
Loss of ribosomal gene function in cardiomyocytes (CMs) invokes TP53-stress response. (**A**) RNA-sequencing of hPSC-CMs following siRNA treatment for *RPL39* and *RPS15A* shows both convergent and divergent transcriptomic response. (**B**) Gene ontology (GO) term analysis of differentially expressed genes following RP KD shows TP53-mediated response, including upregulation of *CDKN1A*. (**C, D**) CDKN1A is highly upregulated in CMs following *siRPS15A* treatment. (**E, F**) Reduced CM proliferation upon *RPS15A* KD is mediated by *CDKN1A*/*TP53* and can be rescued upon their co-KD. (**G, H**) Larval zebrafish CM number is reduced by morpholino treatment for rps15a and can be attenuated by P53 morpholino co-injection. Control and morphant (MO) hearts of 72 hpf zebrafish larva stained with *Tg(myl7:EGFP)* and *Tg(myl7:H2A-mCherry)*. Note that the smaller heart with aberrant looping by *rps15a* MO is partially reversed by *p53* co-KD. Student’s *t*-test, *p < 0.05, **p < 0.01, ***p < 0.001, ****p < 0.0001.

To investigate how RPs regulate cell cycle activity in hPSC-CMs, we performed RNA-seq upon KD of *RPS15A* and *RPL39*, which belong to the small and the large ribosomal subunits that harbor predicted-damaging variants in 75H and 151H probands, respectively. Comparison of differential gene expression between *siRPS15A* and *siRPL39* KD revealed that ~53% of genes were commonly dysregulated (Figure 4A), indicating the existence of a conserved RP-dependent transcriptional network. Consistent with a central role for RPs in the regulation of proliferation, GO term analysis for differentially expressed genes revealed an enrichment for genes involved in DNA replication, mitosis, and DNA damage response mediated by p53, and included the downregulation of positive regulators of cell cycle such as *CDK1*, *CCNA2*, *AURKA*, *PCNA*, and the upregulation of cell cycle arrest mediators such as *CDKN1A*, *BTG2*, *GADD45A* (Figure 4B and Supplementary file 5 and 6). To confirm these findings, we performed immunostaining for canonical p53 downstream transcriptional target (Fischer, 2017), *CDKN1A*, and observed that the percentage of CDKN1A+ CMs was increased by ~60% in response to *RPS15A* KD (Figure 4C, D), indicating that RP KD induces cell cycle arrest *via* the activation of the p53 signaling pathway in hPSC-CMs.

Next, to evaluate if the reduction in proliferation caused by RP KD is mediated by the p53 signaling pathway in CMs, we co-KD *TP53* or *CDKN1A* along with *RPS15A* and observed a significant rescue of CM proliferation by EdU incorporation and CM number in comparison to RPS15A KD (Figure 4E, F and Figure 3—figure supplement 3D, E).

Next, we tested if RP-mediated defects in zebrafish can also be rescued by inhibition of p53. To this aim, we used MOs to co-KD *p53* with *rps15a* (Robu et al., 2007) and examined larval hearts at 72 hpf. *p53* MO-mediated KD on its own had little effect on heart function but partially reversed the *rps15a* MO-mediated reduction in heart size and CM numbers (Figure 4G, H). Moreover, bradycardia, heart looping, and reduced contractility phenotypes of *rps15a* morphants were also rescued by co-KD of *p53* (Figure 3—figure supplement 3F, G). Collectively, these observations support our hypothesis that RPs control cardiac development, including heart size and function, via a p53 pathway-mediated regulation of CM proliferation.

### Heart loss in *Drosophila* by RpS15Aa KD is partially rescued by Hippo pathway activation

The fly heart develops similarly to the vertebrate heart at early stages (Bodmer, 1995) and a link between RPs and cell cycle-regulating pathways, such as p53 and Hippo pathways (Furth et al., 2018), has been proposed to be conserved between mammals and flies (Baker et al., 2019). To test whether p53 has a function in fly heart development similar to hPSC-CMs and zebrafish, we performed cardiac co-KD of *RpS15Aa* and *p53* and examined heart structure and function. Co-KD did not rescue the cardiac defects of *RpS15Aa* KD, suggesting that *p53* has a different role in flies, as compared to vertebrates, as has been reported previously (Ollmann et al., 2000).

**Figure 5.**
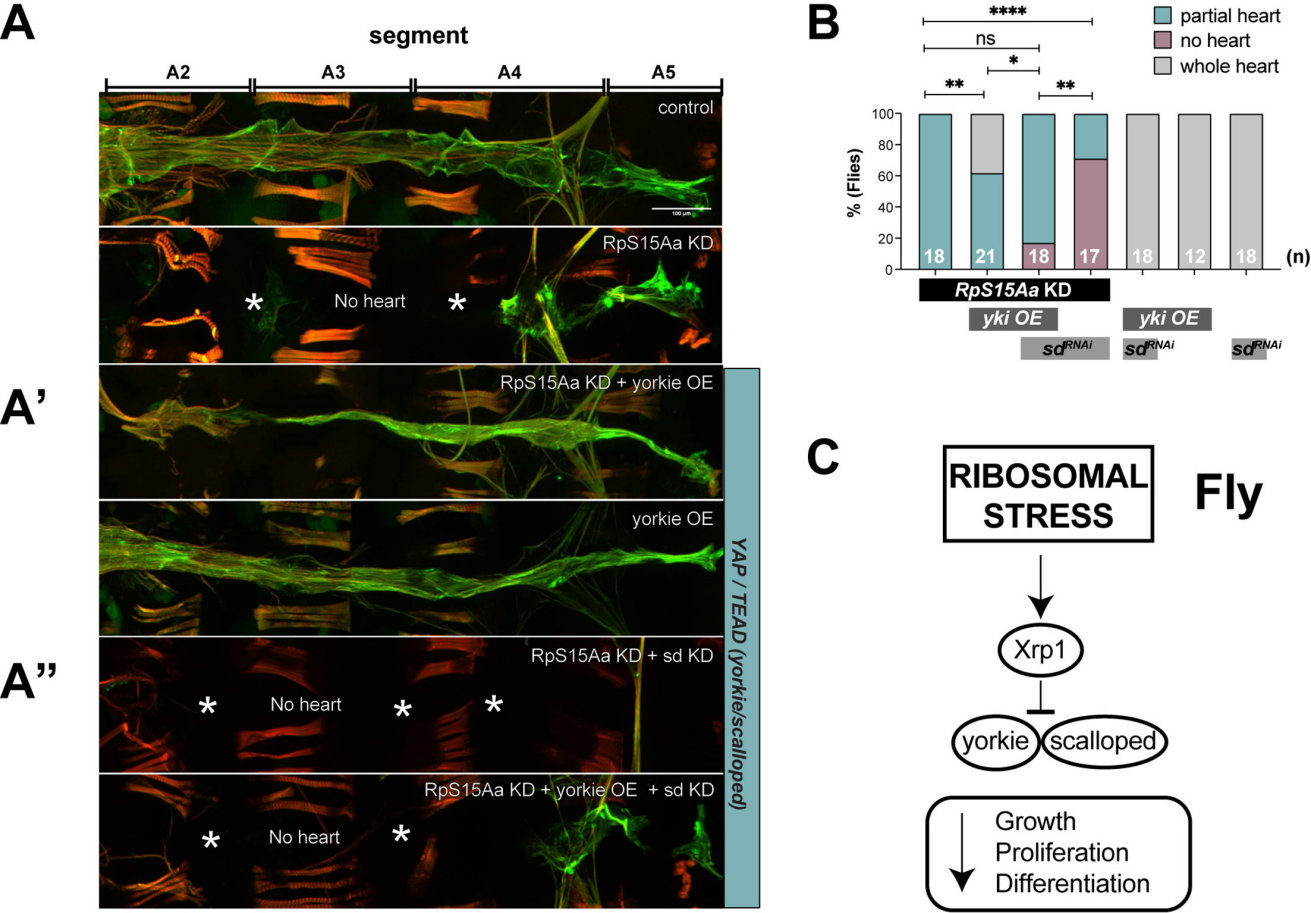
Rescue of *RpS15Aa* KD-mediated heart tube loss in *Drosophila* by YAP/yorkie overexpression depending on its co-factor TEAD/scalloped. (**A**) Representative images of RFP-expressing fly hearts. RpS15Aa KD-mediated heart tube loss can be partially rescued by overexpression of *yorkie* (*RpS15Aa* RNAi + *yorkie* OE). The rescue by *yki* OE depends on its co-factor *sd*. Flies were raised at 25°C. (**B**) Quantification of events presented as a percentage of flies exhibiting whole heart tube versus partial heart loss (defined as 25–75% heart tube length compared to wildtype) or no heart tube. Statistics: Fisher’s exact test, *p < 0.05. (**C**) Proposed signaling cascade underlying cardiac growth, proliferation, and differentiation impairment following ribosomal stress (adapted from Baker et al., 2019).

In vertebrates, TP53 is known to act through the negative regulation of the Hippo pathway (Raj and Bam, 2019). In this context, YAP, a downstream effector of Hippo, has been shown to promote CM proliferation in the mouse heart (Monroe et al., 2019). We therefore tested if YAP/yorkie can substitute the function of p53 in the fly heart and found that overexpressing the Hippo pathway gene *yorkie* (*yki*), the fly ortholog of YAP, in the developing heart considerably restored *RpS15Aa* KD-induced heart loss in *Drosophila* (Figure 5A’, B). We also observe the formation of adult ostial structures, which indicates that the larval heart underwent partial remodeling to an adult heart during metamorphosis. Further, we found that *yki*-mediated rescue depends on the transcriptional co-factor encoded by *scalloped* (*sd*, *Drosophila TEAD1/2/3/4*), since upon *sd* KD, *yki* OE can no longer rescue the *RpS15Aa* KD-induced heart loss (Figure 5A”, B). Moreover, KD of *sd* in an *RpS15Aa* KD background (without *yki* OE) worsens the *RpS15Aa* KD phenotype (Figure 5A”, B).

### *RPS15A* genetically interacts with cTFs *nkx2.7*/*tinman*, and TBX5/*tbx5a/Dorsocross*, *nkx2.7*/*tinman*, and *Gata4,5,6/pannier* in model systems

**Figure 6.**
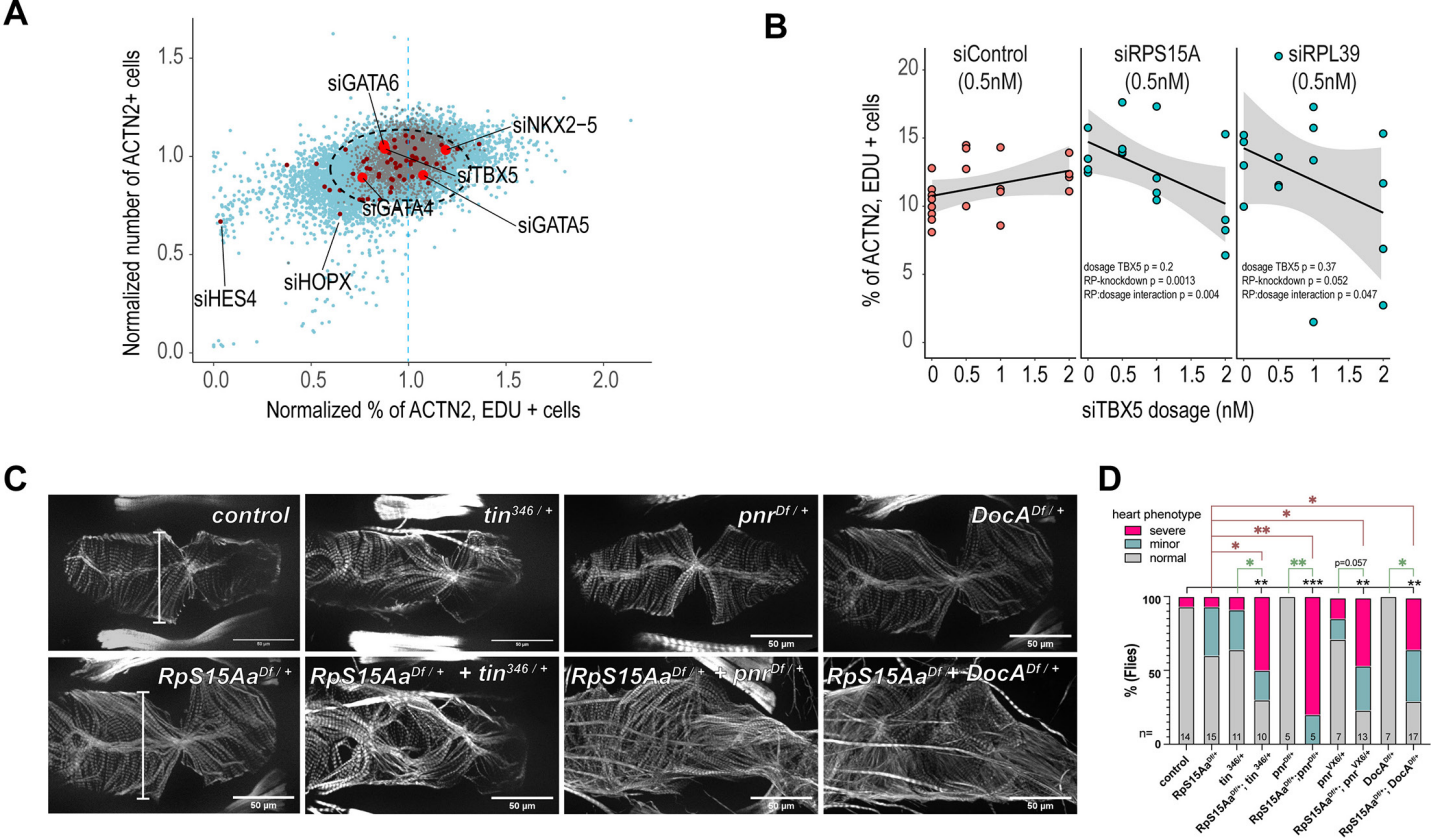
*RPS15A* genetically interacts with cardiac transcription factors. (**A**) The majority of cardiac transcription factors do not impact cardiomyocyte (CM) proliferation, except for, e.g., HES4 and HOPX. (**B**) *TBX5* genetically interacts with *RPS15A* and *RPL39. siTBX5* does not impact CM proliferation at 0.5, 1, 1.5, or 2 nM si-concentration, and neither do *siRPS15A* and *siRPL39* alone at 0.5 nM. CM proliferation is reduced in siRP backgrounds with increased titration of *siTBX5*. Two-way ANOVA for si*TBX5* dosage, RP-knockdown, and their interaction. (**C**) Representative fly heart segment (A4) from control flies, heterozygous mutants (*tin*^346/+^, *pnr*^VX6/+^, *Doc*^Df/-^, *Df*(*RpS15Aa*)^+/-^) and transheterozygous mutants. *tin*^+/-^ = *tin^346^*/+, pnr^+/-^ = *Df(pnr)*/+, Doc^+/-^ = *Df(DocA)*/+. Note the deformation and myofibrillar disorganization in the transheterozygous mutants. (**D**) Quantification of adult *Drosophila* heart defects and genetic interaction. Statistics: Fisher’s exact test on absolute numbers testing *normal* versus *severely deformed* hearts. *p < 0.05, **p < 0.005, ***p < 0.001.

The profound importance of RPs for cell growth and proliferation in most cell types (Kang et al., 2021 and Figure 1—figure supplement 1) raises the question of how tissue-specific phenotypes such as those observed in HLHS can arise from the defective function of ubiquitously expressed genes. To address this apparent paradox, we hypothesized that RPs might functionally interact with tissue-specific proteins such as cTFs to control CM proliferation. Analysis of the impact of cTFs alone on hPSC-CM proliferation from our whole-genome screen revealed that KD of most cTFs did not cause major proliferation defects (except for, e.g., *HES4* and *HOPX*; Figure 6A). Among the cTFs, TBX5 is specifically expressed in the left ventricle at stages of intense CM proliferation (Bruneau et al., 1999) and thus we next asked if *TBX5* could functionally interact with RPs. Remarkably, while hPSC-CM proliferation was not affected by increasing doses of siTBX5 (0–2 nM), increasing si*TBX5* dosage in siRP backgrounds led to proportional decrease in EdU incorporation (two-way ANOVA; Figure 6B and Figure 6—figure supplement 1A, B), thereby indicating that *RPs* and *TBX5* genetically interact to regulate proliferation in hPSC-CMs.

**Figure 6—figure supplement 1.**
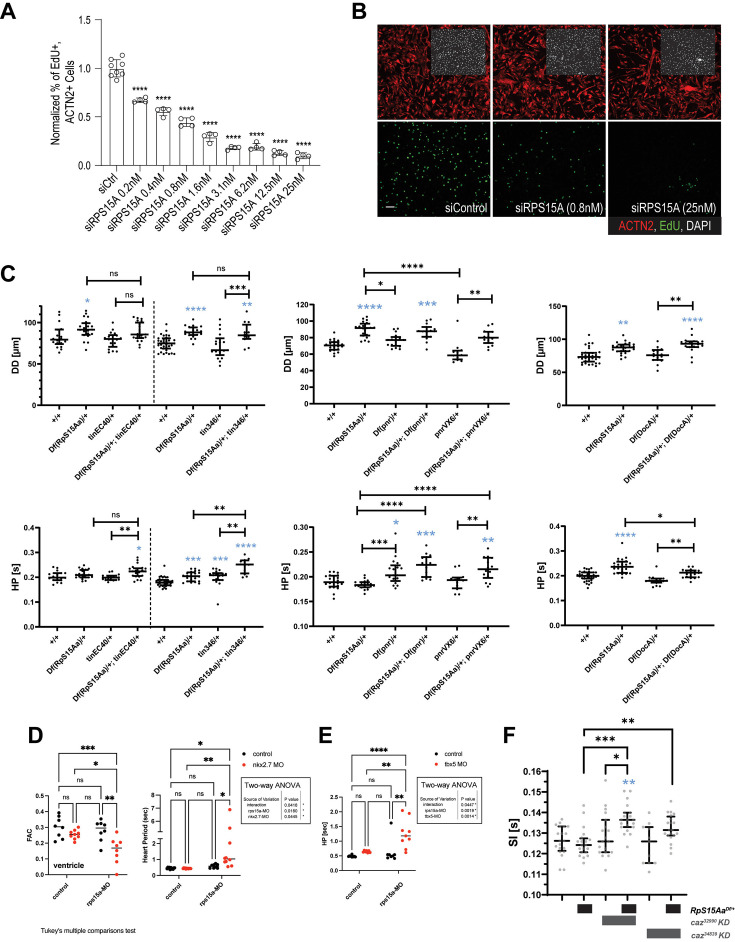
Genetic interaction between *RpS15Aa/RPS15A* and *cabeza/EWSR1*. (**A, B**) Dose-dependent inhibition of hPSC-cardiomyocytes (CMs) proliferation by RPS15A KD. (**C**) Diastolic diameter (DD), and heart period (HP) of 3-week-old female control and heterozygous RpS15Aa deficient flies with or without additional heterozygous mutation in tinman, pannier, or Dorsocross. Statistics: Kruskal–Wallis, *p < 0.05, **p < 0.005, ***p < 0.001, ****p < 0.0001. Note the prolonged HP in transheterozygous mutants for Df(RpS15Aa) and tinman. (**D, E**) FAC and HP of the atria and ventricles at 72 hpf in zebrafish embryos injected with 0.5 ng rps15a and/or 1 ng nkx2.7 MOs (**E**) or 0.5 ng rps15a and/or 0.5 ng tbx5 MOs (**F**). Note that FAC and heart periods show synergistic genetic interaction between rps15a and nkx2.7 and tbx5a. Statistics: Two-way ANOVA, *p < 0.05, **p < 0.01, ****p < 0.0001. (**F**) Systolic intervals (SI) of 1-week-old adult flies upon heart-specific knockdown of *cabeza* (*caz^32990^* or *caz^34839^* RNAi) using Hand^4.2^-Gal4 driver with or without additional RpS15Aa heterozygosity (RpS15Aa^Df/+^) and age-matched controls. Automated analysis of SI depicted as median with interquartile range obtained from 5 s high-frame-rate videos. Statistics: Kruskal–Wallis test, *p < 0.05, **p < 0.01, ***p < 0.001.

Conservation of genetic interactions between human and fly cTFs and constitutive genes has been identified before (Qian et al., 2011). We therefore specifically tested for genetic interaction between *RpS15Aa* and the cardiac TFs *tinman/NKX2–5*, *pannier/GATA4/5/6*, and *Dorsocross/TBX5* in adult *Drosophila* hearts*.* A heterozygous deficiency covering *RpS15Aa* causes moderate dilation of hearts compared to controls (Figure 6—figure supplement 1C). However, when *RpS15Aa* was placed in trans to loss of function alleles of cardiac TFs *tinman*, *pannier*, or *Dorsocross1/2/3*, these hearts exhibited significantly deformed hearts, which is only rarely observed in the single heterozygotes (Figure 5C, D). In addition, *RpS15Aa* heterozygous flies showed a prolonged heart period when combined with a *tinman* or *pannier* heterozygous mutations (Figure 6—figure supplement 1C).

Next, we tested whether similar genetic interactions are conserved in zebrafish. Zebrafish express the NKX2–5 ortholog *nkx2.7* in the heart, and functional studies have shown that both *nkx2.5* and *nkx2.7* play critical roles in cardiac development (Lee et al., 1996). To determine whether *rps15a* and *nkx2.7 or tbx5a* genetically interact in zebrafish, we injected low doses of *rps15a* (Uechi et al., 2006) MO, in combination with *nkx2.7* (Targoff et al., 2008) or *tbx5a* MO. *nkx2.7* MO alone had little effect on heart size, cross-sectional area, or contractility, whereas double morphants exhibited cardiac dysfunction, with contraction virtually abolished in some animals (Figure 6—figure supplement 1D). Furthermore, we observed a significant prolongation of the heart period in double morphants (*rps15a + nkx2.7* or *rps15a + tbx5*a) in comparison to each MO alone (two-way ANOVA; Figure 6—figure supplement 1D, E) consistent with the effects observed in *Drosophila*. Together, these results highlight the existence of evolutionarily conserved (fly, zebrafish, and hPSC-CMs) genetic interactions between RPs and cardiac TF genes in the regulation of cardiogenesis. These data also illustrate how the activity of ubiquitously expressed genes such as RPs can be modulated by cell type-specific genes, like cardiac TFs, to achieve tissue-specific outcomes.

### RP-regulated HLHS-associated genes control proliferation in a CM-specific manner

**Figure 7.**
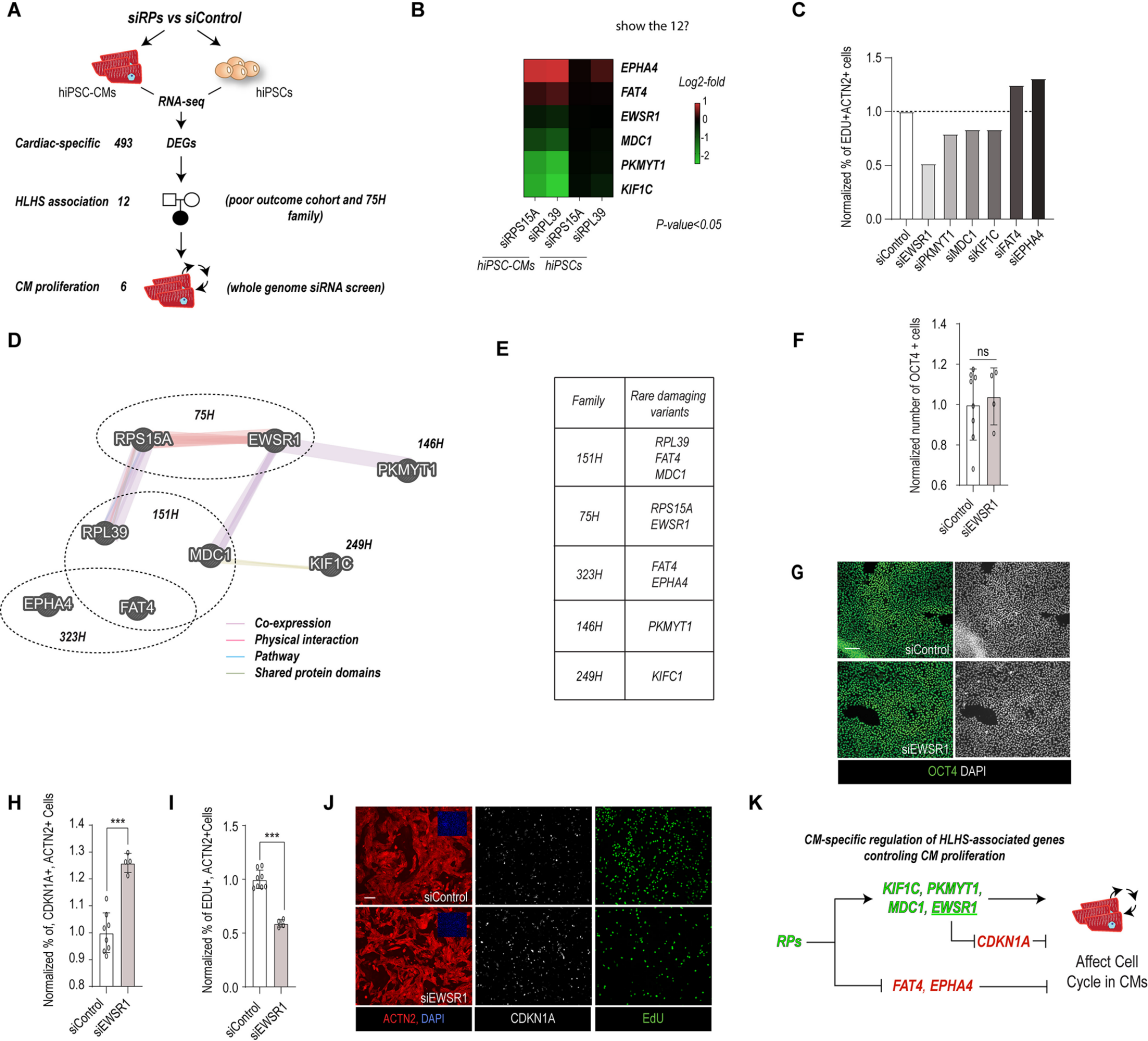
RP-dependent cardiac-specific regulation of cell proliferation. (**A**) Schematic illustrating approach to identify novel RP-dependent and cardiac-specific hypoplastic left heart syndrome (HLHS)-associated gene network controlling cardiomyocyte (CM) proliferation. (**B**) Heatmap showing differential expression (hiPSC-CMs vs hiPSCs) of genes regulating CM proliferation. (**C**) Histogram showing effect of cardiac-specific and HLHS-associated genes on CM proliferation. (**D**) Visualization of RP-dependent and cardiac-specific HLHS-associated gene network (GeneMania). HLHS families. (**E**) Table of HLHS families harboring rare and damaging variant in RP-dependent and cardiac-specific genes. (**F**) Histogram showing lack of effect of siEWSR1 on OCT4+ hiPSCs. (**G**) Representative images of OCT4+ cells in siControl and siEWSR1. (**H**) Histogram showing that siEWSR1 increases the % of CDKN1A+ CMs as compared to siControl. (**I**) Histogram showing that siEWSR1 concomitantly decreases the % of EDU+ CMs as compared to siControl. (**J**) Representative images showing immunostaining for CDKN1A (white), EDU (green), and ACTN2 (red) in siEWSR1 and siControl conditions. (**K**) Pathway reconstruction of cardiac and RP-dependent regulation of CM proliferation by HLHS-associated genes. Chi-square test, *p < 0.05, **p < 0.005, ***p < 0.001.

Previous studies and this work show that reduced RP function can lead to tissue-specific developmental defects, that in turn can be rescued by loss of p53 (Tiu et al., 2021; McGowan et al., 2011). While these findings highlight a downstream role for p53 in the development of RP-dependent phenotypes, they also imply that RPs can regulate cell cycle and/or p53 activity in a tissue-specific manner. Thus, to delineate how RPs might control proliferation in a CM-specific manner, we compared differential gene expression profiles upon the two RP KDs in hPSC-CMs and undifferentiated hPSCs (Figure 7A). Consistent with our hypothesis, this analysis revealed that RPs regulate the expression of 493 genes in a CM-specific manner (Supplementary files 5–8), thereby suggesting the existence of a cell type-specific (cardiac) transcriptional response to RP loss of function.

Next, to evaluate whether HLHS-associated genes were part of this lineage-specific transcriptional response, we selected those differentially expressed genes harboring rare and predicted-damaging variants in probands from the poor-outcome cohort and 75H family (Supplementary files 2 and 4). Remarkably, this prioritization strategy identified 12 genes potentially associated with HLHS (Supplementary file 9), 6 of which were found to also regulate cell cycle activity in hPSC-CMs (Figure 7B, C). Thus, collectively, this approach led us to identify an RP-dependent and cardiac-specific HLHS-associated gene network that supports CM proliferation *via* the upregulation of *EWSR1*, *MDC1*, *PKMYT1*, and *KIF1C*, and downregulation of *FAT4* and *EPHA4* (Figure 7D, E).

**Figure 8.**
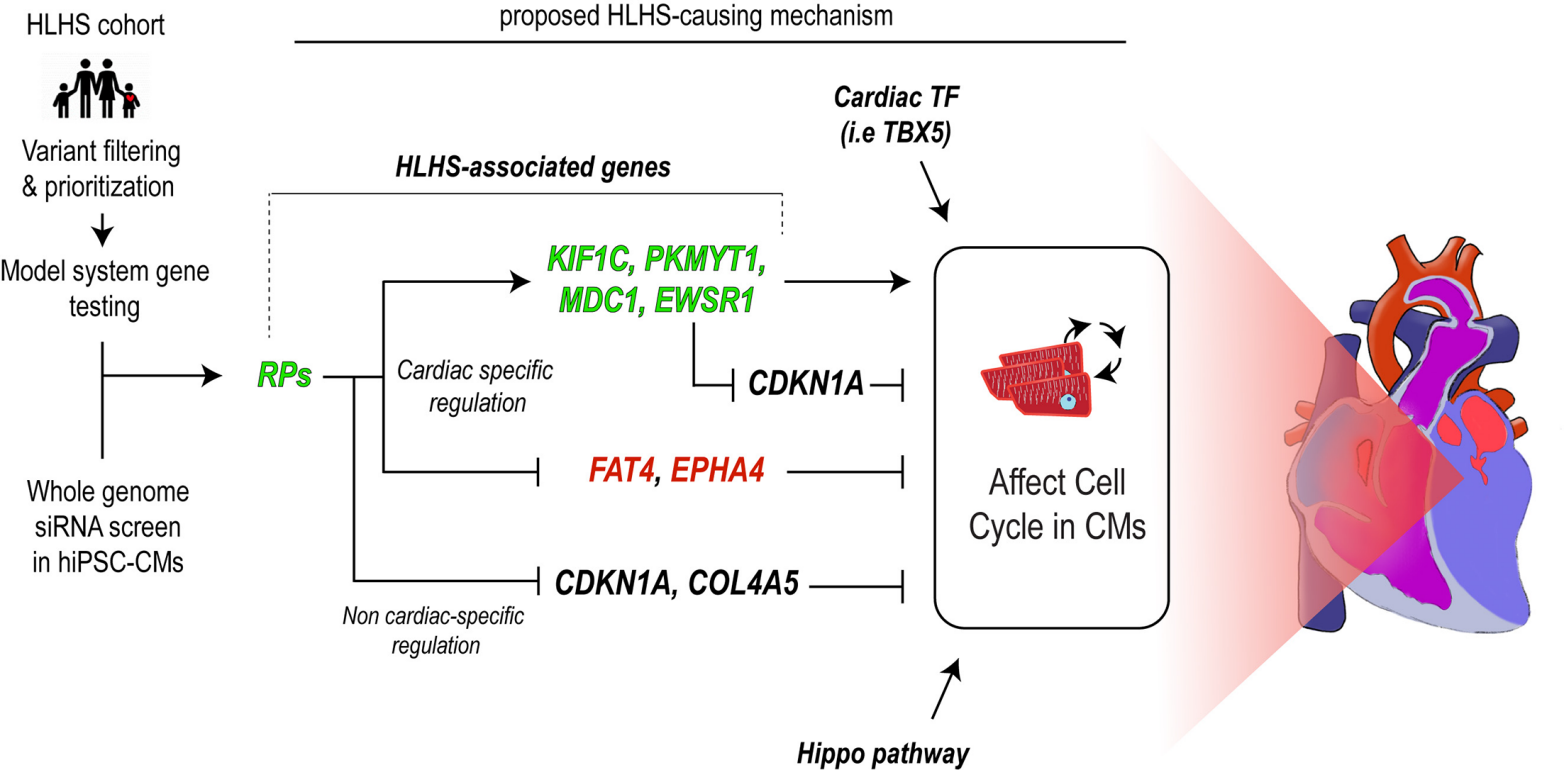
Model for RP-dependent involvement in hypoplastic left heart syndrome (HLHS)-associated phenotypes. Schematic showing that combined prioritization and unbiased screening led to the identification of a novel HLHS-associated gene network regulating cardiomyocyte (CM) proliferation as potential disease-causing mechanism.

To further explore if RP-regulated HLHS-associated genes control proliferation in a cell type-specific manner, we asked if *EWSR1* KD*,* which elicited the strongest proliferation phenotype in hPSC-CMs, could regulate proliferation in non-cardiac cell types such as hPSCs. In contrast to hPSC-CMs, siEWSR1 did not affect the number of OCT4+ cells (Figure 7F, G), which suggests that EWSR1 is able to regulate proliferation CM-specifically. EWSR1 is an RNA/DNA-binding protein that regulates transcription and RNA splicing and plays a major role as oncogenic driver when fused to ETS transcription factors (Flucke et al., 2021). In the next step, we tested if EWSR1 regulates CM proliferation by modulating the p53 pathway. Consistent with this hypothesis, immunostaining for p53 downstream target, CDKN1A, revealed that EWSR1 KD both increased the percentage of CDKN1A-positive CMs and decreased EdU incorporation as compared to siControl (Figure 7H–J). Consistent with these observations, *RpS15Aa* deficiency fly line placed in trans to loss of function allele for *cabeza* (*caz*), the ortholog of *EWSR1*, caused prolongation of systolic interval lengths (Figure 6—figure supplement 1F) as compared to controls, suggesting a genetic interaction between these two genes. Collectively, these results support the conclusion that RPs can modulate proliferation in a cell type-specific manner, by controlling the expression of downstream effectors (i.e. *EWSR1*), with a cell type-specific ability to modulate the p53 pathway activity (Figures 7k and 8).

**Figure 8—figure supplement 1.**
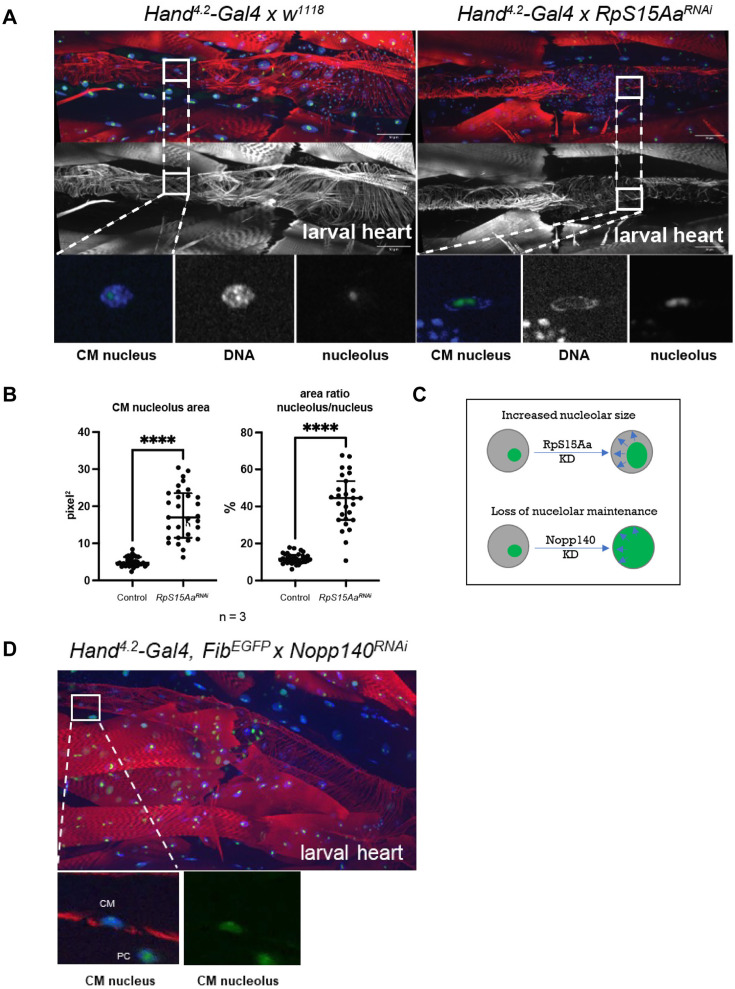
RpS15Aa KD induces nucleolar stress in third instar larval hearts. (**A**) Nucleolar stress induction in third instar larval hearts upon *RpS15Aa* KD as indicated by enlarged nucleoli. Hearts from control and *RpS15Aa* KD. Third instar larva stained for F-actin (red), DAPI (blue), and nucleolus marker Fibrillarin (green) and cardiomyocyte (CM) nuclei in magnifications. Note, heart constriction upon *RpS15Aa* KD in the larval heart. (**B**) Quantification of nucleolus area and area ratio (nucleolus/nucleus) indicates significantly enlarged nucleoli upon *RpS15Aa* KD. Statistics: Mann–Whitney test, ****p < 0001. (**C**) Schematic representation of different nucleolar phenotypes upon RNAi-mediated loss of function. (**D**) As a positive control, KD of another nucleolar key player *Nopp140* specifically in the heart using Hand^4.2^-Gal4 combined with Fibrillarin^EGFP^ (kind gift of the Wieschaus lab) leads to loss of nucleolar maintenance. Third instar larva stained for F-actin (red) and DAPI (blue). Fibrillarin in green. CM nuclei in magnifications. PC = pericardial cell.

## Discussion

### Implication of RPs as genetic modulators in HLHS pathogenesis

Recent work, including our own, strongly suggests that defective cardiac differentiation and impaired CM proliferation contribute to HLHS-associated heart defects (Liu et al., 2017; Gaber et al., 2013; Theis et al., 2020). In this study, we find that RPs are critical for maintaining CM proliferation, as well as cardiac structure and function in fish and fly heart models. We show a potential role for RP function in cardiac development and HLHS pathogenesis as: (1) RPs are central regulators of CM proliferation; (2) RP variants are enriched in a cohort of poor-outcome HLHS patients; (3) a rare predicted-damaging promoter variant in *RPS15A* was associated with HLHS in familial CHD (75H); (4) KD of RPs in vivo results in cardiac deficiencies in hPSC-CMs, *Drosophila*, and zebrafish; and (5) induces TP53 signaling and blocks proliferation that can be alleviated by inhibition of TP53 in the vertebrate models. Interestingly, the transcriptomic response of a previously reported HLHS proband (Theis et al., 2020) paralleled these findings and raised the possibility of an underlying ribosomopathy condition in that HLHS family. While universally important for cellular growth, an increasing number of variants in RP genes have been linked to CHD in humans, indicating tissue-specific differences in the penetrance of RP gene mutations. Most notably, ~30% of patients with Diamond-Blackfan anemia (Vlachos et al., 2018), a ribosomopathy characterized by hypo-proliferative, proapoptotic erythropoiesis, also have CHD. In agreement with another study (Ikeda et al., 2017), *rps15a* KD in fish caused a noticeable reduction in circulating red blood cells.

In addition to RPS15A, *RpL13* was recently identified as a potential candidate gene involved in CHD from a screen for de novo copy number variations in 167 patients with CHD (Schroeder et al., 2019). Furthermore, the Pediatric Cardiac Genomics Consortium (PCGC) exome dataset of 2871 patients with CHD identified several rare, predicted-damaging de novo variants in RP genes, 2 of which were found in patients with HLHS (Jin et al., 2017). Our finding that RPs genetically interact with core cTFs provides a potential mechanism for the cardiac specificity of RP phenotypes. In this context, we hypothesize that RP gene variants might play a role as genetic modifiers (or sensitizers) in the oligogenic pathogenesis of HLHS.

### Cardiac-specific and RP-dependent regulation of proliferation in the context of HLHS

Defective proliferation in LV CMs is a phenotypic hallmark of HLHS (Liu et al., 2017; Gaber et al., 2013), thereby implying the existence of HLHS-associated mechanisms that regulate proliferation in a CM-specific manner. In this study, we identified RPs as novel candidate genetic regulators of hypoplastic phenotypes observed in HLHS, as (1) rare, predicted-damaging variants affecting RP genes are enriched in HLHS probands as compared to healthy controls, (2) KD of most RPs impairs proliferation in hiPSC-CMs (59/80 tested) and heart development in flies (6/6 tested), and (3) systemic loss of *rps15A* function causes heart-specific hypoplastic phenotypes in zebrafish. However, in this context, the evaluation of RPs function in multiple cell types revealed that they are generally required for proliferation (hPSCs, hPSC-CMs, and dermal fibroblasts), thus indicating that RP loss of function triggers a non-cell type-specific cell cycle block.

Thus, an appealing mechanism by which RP function could be invoked in a spatially restricted manner would involve the regulation of RP expression by cell type-specific TFs. Consistent with this model, all probands harboring RP variants contain mutations affecting their promoter regions (Table 1). Also, several of these mutations are predicted to disrupt the binding site of previously identified HLHS-associated TF, ETS1 (Miao et al., 2020; Ye et al., 2010). Consistent with these observations, a recent study from the Bruneau lab (Kathiriya et al., 2021) shows that 38 RP genes are downregulated in TBX5-null hiPSC-CMs and ChIP analysis (Ang et al., 2016) reveals that a third of these dysregulated RPs (14/38 genes) are bound by TBX5 at their core promoter regions. Thus, we speculate that cell type-specific TFs controlling RP expression represent a promising gene class enabling the modulation of RP-dependent proliferation in a tissue-specific manner.

A second mechanism by which RPs might control proliferation and differentiation in a CM-specific manner is based on the hypothesis that CMs may be molecularly more sensitive to RP loss of function than other cell types. Consistent with this model, RP deficiency predisposes flies, fish, and hPSC-CM for interactions with core cardiogenic TFs, including TBX5/*Doc*, GATA4/*pnr*, and NKX2–5/*tin*, the latter being the most cardiac restricted. (Figure 6). Moreover, our comparative transcriptomics analysis (Figure 4) revealed that RPs regulate gene expression in a CM-specific manner. Among these RP-regulated genes, *EWSR1*, *MDC1*, *PKMYT1*, *KIF1C*, *FAT4*, and *EPHA4*, were found to be mutated in HLHS families and regulate CM proliferation (Figure 7). Remarkably, EWSR1 KD did not affect proliferation in hPSCs, while it strongly reduced EdU incorporation in hPSC-CMs, thus suggesting that EWSR1 regulates proliferation in a CM-specific manner. Interestingly, in flies, *RpS15Aa* genetically interacts with *cabeza* (*caz*, the fly ortholog of *EWSR1*). Consistent with our observations, a recent study has found that overexpression of a fusion Ewsr1-Fli1 protein in mice specifically induces dilated cardiomyopathy by promoting apoptosis in cardiac myocytes (Tanaka et al., 2015). Similarly, Fat4, a negative regulator of the Hippo pathway, which is upregulated upon RP KD, was found to specifically regulate heart size via the regulation of CM size and proliferation (Ragni et al., 2017). Thus, collectively, we propose that RPs represent a central regulatory hub for cell proliferation and differentiation during embryonic development, which can be modulated in a cell type-specific manner by tissue-restricted modulators of RP expression and/or activity.

### An RP–MDM2–p53/YAP surveillance network in heart development

Defects in ribosome biogenesis caused, for example, by reduction in RP gene copy numbers induce a stress response program that activates TP53 (p53) and a cascade of cellular events resulting in apoptosis, inhibition of the cell cycle, or DNA damage response (reviewed in e.g. Danilova and Gazda, 2015), commonly referred to as nucleolar stress response (Boulon et al., 2010) with activation of TP53 being a key hallmark (Yang et al., 2018). A key mechanism involves the release of RPs to the nucleoplasm, where several RPs bind and inhibit MDM2, which again results in p53 stabilization and pathway activation (Dai et al., 2004; Zhang and Lu, 2009). A recent study showed that RP haploinsufficiency in the developing mouse limb bud also activates a common TP53 cascade but results in TP53-dependent tissue-specific changes of the translatome, which might confer the specificity often observed in ribosomopathy (Tiu et al., 2021). The TP53-MDM2 feedback loop is the central molecular node in response to a wide variety of stress signals, including in the human diseased adult heart (Men et al., 2021; Mak et al., 2017). As mentioned above, the transcriptomic response of the HLHS family 5H (Theis et al., 2020) also showed activation of the TP53 pathway potentially due to an underlying ribosomopathy. Since low RP levels induce a proliferation blockade that can be overridden by p53-p21/CDKN1A KD, we hypothesize that RP levels may act as signaling components sensing cellular fitness and with a functional outcome either being p53 activation or non-activation. Such an RP–MDM2–p53 surveillance network was previously proposed to be important in response to nutrient availability changes and inhibition of oncogenic activity (Deisenroth and Zhang, 2011; Liu et al., 2016). Thus, the inhibition of the RP–MDM2–p53 axis might be a therapeutic avenue to consider for functional intervention, although further studies are needed to identify the upstream mechanisms leading to the *p53* pathway activation observed in HLHS proband cells.

Heart-specific KD of the *RpS15Aa* in *Drosophila* causes constriction of larval hearts and atrophy in adult hearts, due to heart loss during metamorphosis, which could not be rescued by p53 reduction, unlike in hPSC-CMs and zebrafish. This might be due to a different, context-dependent role of *p53* in flies previously reported to cause a dwarfing, *Minute*-like phenotype (Cui and DiMario, 2007). We hypothesized that the loss of RPs in flies, in conjunction with reduced protein synthesis, might cause nucleolar stress, triggering a cell-intrinsic signaling cascade that prevents the heart from further differentiating and growing. To determine whether cardiac KD of *RpS15Aa* causes nucleolar stress in the *Drosophila* heart, we stained larval hearts for Fibrillarin, a marker for nucleoli and nucleolar integrity. We found that *RpS15Aa* KD causes expansion of nucleolar Fibrillarin staining in CM, which is a hallmark of nucleolar stress (Figure 8—figure supplement 1A–C). As a control, we also performed cardiac KD of *Nopp140*, which is known to cause nucleolar stress upon loss of-function. We found a similar expansion of Fibrillarin staining in larval CM nuclei (Figure 8—figure supplement 1C, D). This suggests that *RpS15Aa* KD indeed causes nucleolar stress in the *Drosophila* heart, which likely contributes to the dramatic heart loss in adults.

The role of the Hippo pathway to regulate cell growth/organ size, proliferation, and survival, and its importance in cardiac biology is conserved between humans and fly (Barron and Kagey, 2014; Del Re, 2014; Yu et al., 2015; Wang et al., 2018). The Hippo–YAP pathway is a cell-intrinsic pathway that regulates CM proliferation and thus heart size during development, as demonstrated by the ability of activated Yap to induce postnatal CM regeneration (Xiao et al., 2016). Interestingly, we find here that overexpression of the *Drosophila* ortholog of YAP/TAZ, Yorkie, rescues *RpS15Aa* KD-induced heart loss, dependent on its downstream factor *scalloped* (the *Drosophila* ortholog of *TEAD1/2/3/4*) (Figure 5). Of note, YAP1 is not only an important regulator of CM proliferation in the embryo but also promotes CM survival and growth in the postnatal heart (von Gise et al., 2012; Del Re et al., 2013), which is in line with our findings. Interestingly, a proband among the PCGC HLHS patients was transheterozygous for mutations in *RPL15* and *TEAD4* (Jin et al., 2017), making this an interesting disease candidate pair.

### Rapid gene discovery and prioritization approach for complex genetic diseases

Uncovering the genetic basis of polygenic and heterogeneous diseases, such as HLHS, remains a significant challenge. This difficulty arises from the lack of experimental approaches capable of rapidly determining the role and contribution of genetic variants, as well as their epistatic relationships, in generating disease-associated phenotypes. The current state-of-the-art approach involves using CRISPR-mediated editing or correction of gene variants (Gifford et al., 2019), which is effective for analyzing single-family pedigrees. However, this method is impractical for prioritizing the large number of candidate genes identified through cohort-wide analyses. Consequently, progress in gene discovery for HLHS has been limited in recent years (Yagi et al., 2018; Birla et al., 2022). In this study, we aimed to address these limitations by employing a HT and unbiased exploration of genes regulating a conserved HLHS-relevant phenotype (Gaber et al., 2013; Miao et al., 2020; Theis et al., 2020; Xu et al., 2022), such as reduced CM proliferation, in hiPSCs. Notably, results integration from a whole-genome functional screen in hiPSC-derived CMs (Figure 1, RPs are top hits from the screen), genomic data from an HLHS parent–proband cohort (Figure 2, RPs represent the most enriched gene class containing rare damaging variants), hiPSC-CM phenotyping from a high-value HLHS family (75H), and functional validation in two independent in vivo model systems (Figure 3), identified RPs as a novel class of HLHS-associated genes. While our study highlights the potential of this approach for gene prioritization, additional research is needed to directly demonstrate the functional consequence of the identified genetic variants, to verify an association between RP encoding genes and HLHS in other patient cohorts with and without poor outcome, and determine if RP variants have a broader role in CHD susceptibility. In conclusion, we propose that the approach outlined in this study provides a novel framework for rapidly prioritizing candidate genes and systematically testing them, individually or in combination, using a CRISPR/Cas9 genome-editing strategy in mouse embryos (Cunningham et al., 2017). Applied in the context of complex genetic diseases, this framework has the potential to yield deeper insights into their underlying mechanisms.

## Materials and methods

**Key resources table:** 

Reagent type (species) or resource	Designation	Source or reference	Identifiers	Additional information
Genetic reagent (*D. melanogaster*)	*Hand^4.2^*-Gal4	Bodmer lab	PMID:16467358	
Genetic reagent (*D. melanogaster*)	R94C02::tdTomato	N. Jan lab	FBtp0137272	
Genetic reagent (*D. melanogaster*)	UAS-RpS15Aa^RNAi^	Vienna *Drosophila* Resource Center (VDRC)	FBgn0010198	v19198
Genetic reagent (*D. melanogaster*)	Df(RpS15Aa)	Bloomington *Drosophila* Stock Center (BDSC)	FBab0047266	39614
Genetic reagent (*D. melanogaster*)	UAS-RpL26^RNAi^	Vienna *Drosophila* Resource Center (VDRC)	FBgn0036825	v40402 v100280
Genetic reagent (*D. melanogaster*)	UAS-RpL36A^RNAi^	Vienna *Drosophila* Resource Center (VDRC)	FBgn0031980	v108391
Genetic reagent (*D. melanogaster*)	UAS-RpS15^RNAi^	Vienna *Drosophila* Resource Center (VDRC)	FBgn0034138	v35415 v104439
Genetic reagent (*D. melanogaster*)	UAS-RpL39^RNAi^	Vienna *Drosophila* Resource Center (VDRC)	FBgn0023170	v23578 v108821
Genetic reagent (*D. melanogaster*)	UAS-RpL3^RNAi^	Vienna *Drosophila* Resource Center (VDRC)	FBgn0020910	v109820
Genetic reagent (*D. melanogaster*)	UAS-yorkie	Pan		
Genetic reagent (*D. melanogaster*)	UAS-Myc^RNAi^	Bloomington *Drosophila* Stock Center (BDSC)	FBgn0262656	25784
Genetic reagent (*D. melanogaster*)	UAS-sd^RNAi^	Vienna *Drosophila* Resource Center (VDRC)	FBgn0003345	v101497
Genetic reagent (*D. melanogaster*)	*Df(3L)DocA*	Reim	Fbab0037663	
Genetic reagent (*D. melanogaster*)	tin^EC40^	Bloomington *Drosophila* Stock Center (BDSC)	Fbal0032861	78560
Genetic reagent (*D. melanogaster*)	tin^346^	Bloomington *Drosophila* Stock Center (BDSC)	Fbal0035787	92964
Genetic reagent (*D. melanogaster*)	pnr^VX6^	Bloomington *Drosophila* Stock Center (BDSC)	Fbal0032468	6334
Genetic reagent (*D. melanogaster*)	Df(pnr)	Bloomington *Drosophila* Stock Center (BDSC)	Fbab0038315	7982
Strain, strain background (*Danio rerio*)	Oregon AB wild-type	Ocorr lab, SBP		A commonly used wild-type strain
Strain, strain background (*Danio rerio*)	*Tg(myl7:EGFP)^twu277^*	Tsai Lab, National Taiwan University	PMID:12950077	A transgenic line of zebrafish labeled with heart-specific EGFP fluorescence
Strain, strain background (*Danio rerio*)	*Tg(myl7:H2A-mCherry)^sd12^*	Yelon Lab, University of California, San Diego	PMID:24075907	A transgenic line of zebrafish specifically expressing mCherry in cardiomyocyte nuclei
Antibody	Mouse monoclonal anti-ACTN1	Sigma	A7811	1:800
Antibody	Mouse monoclonal anti-POU5F1 (OCT4)	Sigma	P0082	1:500
Antibody	Donkey polyclonal anti-mouse Alexa Fluor 568	Invitrogen	A10037	1:500
Antibody	Chicken polyclonal anti-GFP	Aves Labs	GFP-1020	1:300
Antibody	Rabbit polyclonal anti-mCherry	Rockland	600-401P16S	1:200
Antibody	Donkey polyclonal anti-chicken AlexaFluor 488	Jackson ImmunoResearch	703-545-155	1:200
Antibody	Donkey polyclonal anti-rabbit AlexaFluor 568	Invitrogen	A10042	1:200
Other	DAPI (iPSC) 500 mg/ml	Sigma	D9542	Nuclear stain 1:1000
Antibody	Mouse anti-Mhc (*Drosophila*)	DSHB	3E8-3D3	1:50
Antibody	Anti-mouse-Alexa Fluor 488	Jackson Labs	115-545-003	1:500
Antibody	Alexa Fluor 647 phalloidin	Invitrogen	A22287	1:500
Other	DAPI (Zebrafish) 500 mg/ml	Invitrogen	D1306	Nuclear stain 1:200
Sequence-based reagent	RPS15A siRNA	Entrez Gene ID: 6210	Dharmacon	On-Target plus, individual sequence
Sequence-based reagent	TP53 siRNA	Entrez Gene ID: 7157	Dharmacon	On-Target plus, Individual Sequence
Sequence-based reagent	CDKN1A siRNA	Entrez Gene ID: 1026	Dharmacon	On-Target plus, SmartPool
Sequence-based reagent	TP53	371502118c1	IDT Integrated DNA Technologies, Coralville, IA	Expression level
Sequence-based reagent	CDKN1A	310832423c1	IDT Integrated DNA Technologies, Coralville, IA	Expression level
Sequence-based reagent	CCNB1	356582356c1	IDT Integrated DNA technologies, Coralville, IA	Expression level
Sequence-based reagent	CCNB2	332205979c1	IDT Integrated DNA technologies, Coralville, IA	Expression level
Sequence-based reagent	CDK1	281427275c1	IDT Integrated DNA technologies, Coralville, IA	Expression level
Sequence-based reagent	MCM2	33356546c1	IDT Integrated DNA technologies, Coralville, IA	Expression level
Sequence-based reagent	RPS15A	71772358c2	IDT Integrated DNA Technologies, Coralville, IA	Expression level
Sequence-based reagent	GAPDH	Hs.PT.45.8326	IDT Integrated DNA Technologies, Coralville, IA	Expression level
Commercial assay or kit	EdU	Click-it Plus EdU Imaging Kit	Life Technologies	
Software, algorithm	Prism v7 and v8	SBP license	GraphPad Software	

### Study subjects

Written informed consent was obtained for HLHS probands and family members under a research protocol approved by the Mayo Clinic Institutional Review Board. The studies described in this manuscript were conducted according to the guidelines of the Declaration of Helsinki and approved by the Institutional Review Board of the Mayo Clinic (HLHS protocol 11-000114 approved 10 March 2011).

Cardiac anatomy was assessed by echocardiography. Candidate genes were identified and prioritized by WGS of genomic DNA and RNA-sequencing of patient-specific iPSC and CMs. Methods for genomic analyses, RNA-sequencing, iPSC technology, bioinformatics, and statistics are described in the Online Appendix.

### WGS and bioinformatic strategies

Our methods for family phenotyping, WGS, variant filtering, and candidate gene prioritization in the rare HLHS-CHD family and 25 HLHS proband–parent trios have been previously described (Theis et al., 2015a; Theis et al., 2015b; Bodmer, 1993). In brief, WGS of DNA isolated from blood or cheek swabs was performed on a HiSeq 4000 platform at the Mayo Clinic Medical Genome Facility. BAM files underwent primary and secondary analyses using an established workflow with standard data quality metrics, and reads were aligned to the human hg38 reference genome. Variant call format files with single nucleotide variants and small insertion–deletion (indel) calls were analyzed using Ingenuity Variant Analysis software and knowledge database (QIAGEN). To retain only high-confidence data, variants were required to have a base call quality of at least 20 and to pass Variant Quality Score Recalibration. Functionally annotated coding and regulatory variants underwent primary filtering and prioritization by an iterative approach with variants required to have an MAF <0.01 across all races in gnomAD v2.1 (Karczewski et al., 2020). Variants predicted to impact protein structure were retained and included missense, frameshift, stop-gain, stop-loss, canonical splice, and non-canonical variants predicted to disrupt splicing based upon MaxEntScan (Grodecká et al., 2014). Regulatory variants were defined as those that (a) impact a microRNA or microRNA-binding site, (b) reside in an enhancer binding site annotated in the VISTA database (http://enhancer.lbl.gov/), or (c) disrupt a predicted promoter or transcription factor-binding site informed by the position-weighted matrices available in JASPAR (http://jaspar.cgb.ki.se/). Genes harboring a rare functional variant were further required to have upper quartile cardiac expression during mouse embryonic (e14.5) or human fetal heart development ranked percentiles from RNA-seq experiments available in ENCODE: ENCSR047LLJ (120-day male); ENCSR863BUL (91-day female); ENCSR000AEZ (28- and 19-week female).

For the HLHS-CHD family, a secondary segregation filter was applied to model autosomal dominant inheritance with incomplete penetrance, which required sharing of prioritized rare variants between affected family members. For the poor-outcome HLHS proband–parent trios, a secondary Mendelian filter was applied to identify major-effect driver variants that arose de novo or fit recessive modes of inheritance (i.e., homozygosity, hemizygosity, compound heterozygosity).

To determine whether certain gene networks were over-represented, two online bioinformatics tools were used. First, STRING (Szklarczyk et al., 2019) was used to investigate experimental and predicted protein–protein and genetic interactions, and clustering of RP genes was demonstrated when the highest stringency filter was applied. In addition, PANTHER (Mi et al., 2019) was employed to identify potential enrichment of genes by ontology classification.

### Generation of hPSC-VCMs

Id1 overexpressing hPSCs (derived from Burridge et al., 2015) were dissociated with 0.5 mM EDTA (Thermo Fisher Scientific) in PBS without CaCl_2_ and MgCl_2_ (Corning) for 7 min at room temperature (RT). hPSCs were resuspended in mTeSR-1 media (StemCell Technologies) supplemented with 2 µM Thiazovivin (StemCell Technologies) and plated in a Matrigel-coated 12-well plate at a density of 3 × 10^5^ cells per well. After 24 hr after passage, cells were fed daily with mTeSR-1 media (without Thiazovivin) for 3–5 days until they reached ≥90% confluence to begin differentiation. hPSC-VCMs were differentiated as previously described (Burridge et al., 2015). At day 0, cells were treated with 6 µM CHIR99021 (Selleck Chemicals) in S12 media (Pei et al., 2017) for 48 hr. At day 2, cells were treated with 2 µM Wnt-C59 (Selleck Chemicals), an inhibitor of WNT pathway, in S12 media. Forty-eight hours later (at day 4), S12 media is fully changed. At day 5, cells were dissociated with TrypLE Express (Gibco) for 2 min and blocked with RPMI (Gibco) supplemented with 10% FBS (Omega Scientific). Cells were resuspended in S12 media supplemented with 4 mg/l Recombinant Human Insulin (Gibco) (S12+ media) and 2 µM Thiazovivin and plated onto a Matrigel-coated 12-well plate at a density of 9 × 10^5^ cells per well. S12+ media was changed at day 8 and replaced at day 10 with RPMI (Gibco) media supplemented with 213 µg/µl l-ascorbic acid (Sigma), 500 mg/l BSA-FV (Gibco), 0.5 mM L-carnitine (Sigma), and 8 g/l AlbuMAX Lipid-Rich BSA (Gibco; CM media). Typically, hPSC-ACMs start to beat around day 10. At day 15, cells were purified with lactate media RPMI without glucose, 213 µg/µl L-ascorbic acid, 500 mg/l BSA-FV, and 8 mM sodium-DL-lactate (Sigma), for 4 days. At day 19, media was replaced with CM media.

### siRNA transfection, proliferation assay, and immunostaining in hPSC-VCMs

At day 25 of differentiation, hPSC-VCMs were dissociated with TrypLE Select 10X (Gibco), 10 min and neutralized with RPMI supplemented with 10% FBS. Cells were resuspended in RPMI with 2% KOSR (Gibco) and 2% B27 50X with vitamin A (Life Technologies) supplemented with 2 μM Thiazovivin and plated at a density of 5000 cells/well in a Matrigel-coated 384-well plate. hPSC-VCMs were transfected with siRNA (Dharmacon: ON-TARGETplus, custom RNAi cherry-pick libraries 0.1 nmol). For the whole-genome screening, siRNAs directed to 18,000 human genes were purchased from the Genomic Center Facility at SBP at a final concentration of 25 nM using lipofectamine RNAiMax (Thermo Fisher) and opti-MEM (Gibco). Forty-eight hours post-transfection, cells were labeled with 10 μM EdU (Thermo Fisher). After 24 hr of EdU incubation, cells were fixed with 4% paraformaldehyde for 30 min and blocked in blocking buffer (10% Horse Serum, 10% Gelatin, and 0.5% Triton X-100) for 20 min. EdU was detected according to protocol and cells were stained with cardiac-specific marker ACTN2 (A7811, Sigma, dilution 1/800), secondary antibody Alexa Fluor 568 (Invitrogen, 1/500) and DAPI (1/1000) in Blocking Buffer. Cells were imaged with ImageXpress Micro XLS microscope (Molecular Devices) and custom algorithms were used to quantify % of EdU+ ACTN1+ hPSC-VCMs and the number of ACTN1+ cells. To quantify TP53 levels in hPSC-VCMs, cells were stained with Phospho-p53 (700439, Thermo Fisher, 1/500), counterstained with ACTN1 (anti-mouse A7811, Sigma, or anti-rat ab50599, Abcam), and imaged and quantified with ImageXpress microscope. Whole-genome screening was performed in one replicate. All the other siRNA experiments were performed in quadruplicates.

### RNA-seq and data analysis

hPSCs and hPSC-derived CMs were transfected with 25 nM final concentrations of siRNA against RPS15A, RPL39, and with scrambled control siRNAs as above. Two days after siRNA transfection, RPs KD were verified and confirmed by proliferation assay (see above), and cells were pelleted and resuspended in 500 µl TRIzol reagent followed by total RNA extraction. Library preparation and sequencing of the samples was done at La Jolla Institute of Immunology (La Jolla, CA). FASTQ files were processed using nf-core/rnaseq (version 21.03.0.edge; Ewels et al., 2020). Differential gene expression was determined using R/DESeq2 (Love et al., 2014) and GO term enrichment was done using gprofiler2 (Raudvere et al., 2019). Analysis scripts can be downloaded at https://github.com/gvogler/elife-2025-nielsen-et-al (copy archived at Vogler, 2025). Raw reads and counts tables are available at GEO accession number GSE207658.

### Proliferation assay in parent/proband iPSCs

hPSCs were dissociated as described previously and plated in a Matrigel-coated 384-well plate at a density of 3000 cells per well. Cells were transfected with siRNA at a final concentration of 25 nM using lipofectamine RNAiMax and opti-MEM. After 3 days, cells were labeled with 10 μM EdU for 1 hr. Cells were fixed with 4% paraformaldehyde for 30 min and blocked in blocking buffer for 20 min. EdU was detected according to the protocol and stained with stem cell-specific marker OCT4 (P0082, Sigma, 1/500), secondary antibody Alexa Fluor 568 and DAPI in Blocking Buffer. Cells were imaged with ImageXpress microscope, and % of EdU+ cells and number of OCT4+ cells were quantified.

### Quantitative real-time PCR (RT-qPCR) in parent/proband iPSCs and hPSC-CMs

Total RNA was extracted using TRIzol and chloroform. 1 µg of RNA was converted to cDNA using QuantiTect Reverse Transcription kit (QIAGEN). qRT-PCR was performed using SYBR green (Bio-Rad). Human primer sequences for qRT-PCR were obtained from Harvard Primer Bank: TP53 (Primer Bank ID: 371502118c1), CDKN1A (Primer Bank ID: 310832423c1), CCNB1 (Primer Bank ID: 356582356c1), CCNB2 (Primer Bank ID: 332205979c1), CDK1 (Primer Bank ID: 281427275c1), MCM2 (Primer Bank ID: 33356546c1), RPS15A (Primer Bank ID: 71772358c2). All values were normalized to *GAPDH* (*Primer Bank ID: 378404907c1)*. At least three independent biological replicates were performed for each experiment.

### Statistical analysis

To determine any statistical significance between experimental and control groups in hPSC-VCMs and iPSCs from parent/proband trio, we calculated two-sided p values with Student’s *t*-test using GraphPad Prism 8.1.2 software.

### *Drosophila* heart function studies

*Drosophila* orthologs were determined using the DIOPT database (Hu et al., 2011), and fly stocks were obtained from the Vienna *Drosophila* Resource Center (VDRC) stock center or Bloomington *Drosophila* Stock Center (BDSC) as indicated in the key source table. For in vivo functional heart analysis, we developed a high-throughput method based on genetically modified flies with CM-specific RFP fluorescence. The reporter line by itself or combined with the heart-specific *Hand^4.2^*-Gal4 driver including all cardioblasts/CMs, pericardial cells, and wing hearts throughout development starting at post-mitotic, mid-embryonic stages through adulthood (Han et al., 2006; Tögel et al., 2013; Hallier et al., 2015) was crossed to UAS-lines or mutant flies. For interaction studies, a fly line harboring the fluorescent reporter, *Hand^4.2^*-Gal4 driver, and a deficiency for RpS15Aa was generated. Adult progeny flies were immobilized and exposed to fluorescence light to record 5 s high-frame-rate movies of the beating heart. Movies were analyzed by fully automated quantification of contractility and rhythmicity parameters of the heart (Vogler, 2021). Semi-intact adult fly hearts were filmed and analyzed according to standard protocol (Fink et al., 2009; Cammarato et al., 2015).

### Immunostainings of the fly heart

The immunostaining of fly adult hearts was performed as described previously (Alayari et al., 2009). In short, adult flies were dissected in a semi-intact fashion to expose the heart according to protocol (Fink et al., 2009; Ocorr et al., 2014). Myofibrils were relaxed using 10 mM EGTA followed by fixation in 4% formaldehyde for 15 min. Hearts were washed with PBS + Triton (PBT; 0.03% Triton X-100) and stained using Alexa 647 phalloidin (Life Technologies) 2 hr at RT. For Mhc labeling, hearts were stained using anti-Mhc antibody (1:50, incubation overnight at 4°C) and after three washes with PBT, secondary antibody was applied for 2 hr at RT. Hearts were washed with PBT three times and PBS one time and mounted using ProLong Gold mountant with DAPI (Life Technologies). Heart preparations were imaged with the Imager.Z1 with an Apotome (Carl Zeiss), Hamamatsu Orca Flash 4.0, and ZEN imaging software (Carl Zeiss).

### Zebrafish husbandry

All zebrafish experiments were performed in accordance with protocols approved by IACUC, AUF 22-073. Zebrafish were maintained under standard laboratory conditions at 28.5°C. In addition to Oregon AB wild-type, the following transgenic lines were used: *Tg(myl7:EGFP)^twu277^* (Huang et al., 2003) and *Tg(myl7:H2A-mCherry)^sd12^* (Schumacher et al., 2013).

In zebrafish, gene expression was manipulated using standard microinjection of MO antisense oligonucleotides (Wang et al., 2018). In addition, we performed targeted mutagenesis using CRISPR/Cas9 genome editing (Talbot and Amacher, 2014; Gagnon et al., 2014; Irion et al., 2014), to create insertion/deletion (INDEL) mutations in relevant ribosomal genes (F_0_). Subsequently, zebrafish were raised to 72 hr post-fertilization (hpf), immobilized in low melt agarose, and the hearts were filmed and analyzed according to standard protocol (Fink et al., 2009).

### MO sequence information

All MOs were purchased from Gene Tools, LLC, synthesized at 300 nmol, except zebrafish p53 oligos at 100 nmol.

Zebrafish *rpl13* 5′-UTR MO: TTGTTCACTCCGTCCTTAGCGGAAA
Zebrafish *rps15a* 5′-UTR MO: CGCACCATGATGCCAGTTCTGCAAT
Zebrafish *rpl39* 5′-UTR MO: GGATCGCAATCCGTTCACCACTATG
Zebrafish p53 MO: GCGCCATTGCTTTGCAAGAATTG
Zebrafish Control MO: 5′-CCT CTT ACC TCA GTT ACA ATT TAT A-3′.

### Zebrafish SOHA (semi-automated optical heartbeat analysis)

Larval zebrafish (72 hpf) were immobilized in a small amount of low melt agarose (1.5%) and submerged in conditioned water. Beating hearts were imaged with direct immersion optics and a digital high-speed camera (up to 200 frame/s, Hamamatsu Orca Flash) to record 30 s movies; images were captured using HC Image (Hamamatsu Corp). Cardiac function was analyzed from these high-speed movies using semi-automatic optical heartbeat analysis software (Fink et al., 2009; Ocorr et al., 2009), which for zebrafish quantifies heart period (R–R interval), cardiac rhythmicity, as well as chamber size and fractional area change. All hearts were imaged at RT (20–21°C). Statistical analyses were performed using Prism software (GraphPad). Significance was determined using two-tailed, unpaired Student *t*-test or one-way ANOVA and Dunnett’s multiple comparisons post hoc test as appropriate.

### Zebrafish CM cell counts and cardiac immunofluorescent imaging

To count CMs, we used the expression of H2AmCherry in the nuclei (*Tg(myl7:H2A-mCherry)*) (Schumacher et al., 2013) to qualify as an individual cell, performed the ‘Spot’ function in Imaris to distinguish individual cells in reconstructions of confocal z-stacks. (Zeng and Yelon, 2014; Pradhan et al., 2017). To compare datasets, we used Prism software (GraphPad) to perform Student’s *t*-test with two-tail distribution. Graphs display mean and standard deviation for each dataset.

Whole-mount immunofluorescence was performed as previously described (Theis et al., 2020; Zeng and Yelon, 2014; Pradhan et al., 2017; Alexander et al., 1998) (see also key resources table). Confocal imaging was performed on an LSM 710 confocal microscope (Zeiss, Germany) with a 40x water objective. Exported z-stacks were processed with Imaris software (Bitplane), Zeiss Zen, and Adobe Creative Suite software (Photoshop and Illustrator 2020). All confocal images shown are projection views of partial reconstructions from multiple z-stack slices, except where noted that images are views of a single slice.

For proliferation assays, embryos at 24, 48, and 72 hpf were fixed in 4% paraformaldehyde overnight at 4°C, washed in PBS with 0.1% Tween-20 (PBST). Samples were blocked in 5% normal goat serum in PBST prior to incubation with primary antibodies. Proliferating cells were labeled with anti-phospho-histone H3 (Sigma-Aldrich-H0412 PH3; 1:500) and total nuclei were counterstained with DAPI. Embryos were mounted in ProLong Gold for imaging. Whole-mount hearts were imaged on a Zeiss Apotome microscope at ×10 magnification. Z-stacks were acquired and reconstructed using Fiji/ImageJ. Regions corresponding to atrium, ventricle, atrioventricular canal, and outflow tract were manually segmented. For some experiments, CM nuclei were identified based on Tg(myl7:H2A-mCherry) expression, with DAPI used to quantify total cell counts. For proliferation experiments, DAPI was used to identify cardiac cells, and proliferating cells were identified by PH3 staining. Proliferative indices were calculated as PH3+/DAPI+ ratios from z-stacks analyzed in Fiji/ImageJ using the Cell Counter plugin. Cell counts were performed on at least 10 embryos per condition per timepoint.

### Zebrafish CRISPR/Cas9 experiments

Detailed steps were previously described (Hoshijima et al., 2019) and we followed IDT manufacture instruction for complexes preparation. crRNA:tracrRNA Duplex Preparation:Target-specific Alt-R crRNA (sequence information see below) and common Alt-R tracrRNA were synthesized by IDT and each RNA was dissolved in duplex buffer (IDT) as 100 μM stock solution. Stock solutions were stored at –20°C. To prepare the crRNA:tracrRNA duplex, equal volumes of 100 μM Alt-R crRNA and 100 μM Alt-R tracrRNA stock solutions were mixed together and annealed by heating followed by gradual cooling to RT by manufacture instruction: 95°C, 5 min on PCR machine; cool to 25°C; cool to 4°C rapidly on ice. The 50 μM crRNA:tracrRNA duplex stock solution was stored at –20°C. Preparation of crRNA:tracrRNA:Cas9 RNP complexes: Cas9 protein (Alt-R S.p. Cas9 nuclease, v.3, IDT) was adjusted to 25 μM stock solution in 20 mM HEPES-NaOH (pH 7.5), 350 mM KCl, 20% glycerol, dispensed as 8 µl aliquots, and stored at –80°C. 25 μM crRNA:tracrRNA duplex was produced by mixing equal volumes of 50 μM crRNA:tracrRNA duplex stock and duplex buffer (IDT). We used 5 μM RNP complex. To generate 5 μM crRNA:tracrRNA:Cas9 RNP complexes: 1 μl 25 μM crRNA:tracrRNA duplex was mixed with 1 μl 25 μM Cas9 stock, 2 μl H_2_O, and 1 μl 0.25% phenol red solution. Prior to microinjection, the RNP complex solution was incubated at 37°C for 5 min and then placed at RT. Approximately one nanoliter of 5 μM RNP complex was injected into the cytoplasm of one-cell stage embryos to generate F_0_ larva.

### IDT crRNA sequence information

All crRNAs were purchased from Integrated DNA Technologies, Inc, synthesized at 2 nmol with standard desalting condition.

Dr.Cas9.RPS15A.1.AC: /AltR1/rUrU rGrUrU rGrUrC rArArU rCrUrC rArCrA rGrGrG rGrUrU rUrUrA rGrArG rCrUrA rUrGrC rU/AltR2/
Dr.Cas9.RPS15A.1.AB: /AltR1/rGrC rGrUrA rCrUrA rUrGrA rCrUrU rUrArG rArGrC rGrUrU rUrUrA rGrArG rCrUrA rUrGrC rU/AltR2/
Dr.Cas9.RPS17.1.AC: /AlTR1/rUrGrArCrUrUrCrCrArCrArUrUrArArCrArArGrCrGrUrUrUrUrArGrArGrCrUrArUrGrCrU/AlTR2/
Dr.Cas9.RPS28.1.AA: /AlTR1/rCrUrGrGrGrArArGrArArCrUrGrGrCrUrCrCrCrArGrUrUrUrUrArGrArGrCrUrArUrGrCrU/AlTR2/

## Data Availability

For RNA-sequencing, raw reads and counts tables are available at GEO accession number GSE207658. The following dataset was generated: VoglerG 2022siRPS15A-3NCBI Gene Expression OmnibusGSM6304454

## Additional files

Supplementary file 1. Whole-genome siRNA screen results table.

Supplementary file 2. 292 filtered variants from poor-outcome HLHS probands. MOI – mode of inheritance.

Supplementary file 3. Subset from all 292 genes that cause CM proliferation defects and/or fly heart defects.

Supplementary file 4. Segregating variants of 75H proband.

Supplementary file 5. Differential gene expression lists for siRPL39 and siRPS15A in hPSCs and CMs.

Supplementary file 6. Differential gene expression lists for siRPL39 and siRPS15A in hPSCs and CMs.

Supplementary file 7. Differential gene expression lists for siRPL39 and siRPS15A in hPSCs and CMs.

Supplementary file 8. Differential gene expression lists for siRPL39 and siRPS15A in hPSCs and CMs.

Supplementary file 9. Twelve genes prioritized after RNA-seq and whole-genome sequencing (WGS) analysis.

MDAR checklist

## References

[bib1] Agopian AJ, Goldmuntz E, Hakonarson H, Sewda A, Taylor D, Mitchell LE, Pediatric Cardiac Genomics Consortium* (2017). Genome-wide association studies and meta-analyses for congenital heart defects. Circulation. Cardiovascular Genetics.

[bib2] Alayari NN, Vogler G, Taghli-Lamallem O, Ocorr K, Bodmer R, Cammarato A (2009). Fluorescent labeling of Drosophila heart structures. Journal of Visualized Experiments.

[bib3] Alexander J, Stainier DY, Yelon D (1998). Screening mosaic F1 females for mutations affecting zebrafish heart induction and patterning. Developmental Genetics.

[bib4] Ang YS, Rivas RN, Ribeiro AJS, Srivas R, Rivera J, Stone NR, Pratt K, Mohamed TMA, Fu JD, Spencer CI, Tippens ND, Li M, Narasimha A, Radzinsky E, Moon-Grady AJ, Yu H, Pruitt BL, Snyder MP, Srivastava D (2016). Disease model of GATA4 mutation reveals transcription factor cooperativity in human cardiogenesis. Cell.

[bib5] Baker NE, Kiparaki M, Khan C (2019). A potential link between p53, cell competition and ribosomopathy in mammals and in Drosophila. Developmental Biology.

[bib6] Bakkers J (2011). Zebrafish as a model to study cardiac development and human cardiac disease. Cardiovascular Research.

[bib7] Barron DA, Kagey JD (2014). The role of the Hippo pathway in human disease and tumorigenesis. Clinical and Translational Medicine.

[bib8] Bier E, Bodmer R (2004). *Drosophila*, an emerging model for cardiac disease. Gene.

[bib9] Birla AK, Brimmer S, Short WD, Olutoye OO, Shar JA, Lalwani S, Sucosky P, Parthiban A, Keswani SG, Caldarone CA, Birla RK (2022). Current state of the art in hypoplastic left heart syndrome. Frontiers in Cardiovascular Medicine.

[bib10] Bodmer R (1993). The gene tinman is required for specification of the heart and visceral muscles in Drosophila. Development.

[bib11] Bodmer R (1995). Heart development in Drosophila and its relationship to vertebrates. Trends in Cardiovascular Medicine.

[bib12] Bodmer R, Frasch M, Rosenthal N, Harvey RP (2010). Heart Development and Regeneration.

[bib13] Boulon S, Westman BJ, Hutten S, Boisvert FM, Lamond AI (2010). The nucleolus under stress. Molecular Cell.

[bib14] Brand AH, Perrimon N (1993). Targeted gene expression as a means of altering cell fates and generating dominant phenotypes. Development.

[bib15] Bruneau BG, Logan M, Davis N, Levi T, Tabin CJ, Seidman JG, Seidman CE (1999). Chamber-specific cardiac expression of Tbx5 and heart defects in Holt-Oram syndrome. Developmental Biology.

[bib16] Burridge PW, Holmström A, Wu JC (2015). Chemically defined culture and cardiomyocyte differentiation of human pluripotent stem cells. Current Protocols in Human Genetics.

[bib17] Cammarato A, Ocorr S, Ocorr K (2015). Enhanced assessment of contractile dynamics in Drosophila hearts. BioTechniques.

[bib18] Cripps RM, Olson EN (2002). Control of cardiac development by an evolutionarily conserved transcriptional network. Developmental Biology.

[bib19] Crucean A, Alqahtani A, Barron DJ, Brawn WJ, Richardson RV, O’Sullivan J, Anderson RH, Henderson DJ, Chaudhry B (2017). Re-evaluation of hypoplastic left heart syndrome from a developmental and morphological perspective. Orphanet Journal of Rare Diseases.

[bib20] Cui Z, DiMario PJ (2007). RNAi knockdown of Nopp140 induces Minute-like phenotypes in *Drosophila*. Molecular Biology of the Cell.

[bib21] Cunningham TJ, Yu MS, McKeithan WL, Spiering S, Carrette F, Huang CT, Bushway PJ, Tierney M, Albini S, Giacca M, Mano M, Puri PL, Sacco A, Ruiz-Lozano P, Riou JF, Umbhauer M, Duester G, Mercola M, Colas AR (2017). Id genes are essential for early heart formation. Genes & Development.

[bib22] Dai MS, Zeng SX, Jin Y, Sun XX, David L, Lu H (2004). Ribosomal protein L23 activates p53 by inhibiting MDM2 function in response to ribosomal perturbation but not to translation inhibition. Molecular and Cellular Biology.

[bib23] Danilova N, Gazda HT (2015). Ribosomopathies: how a common root can cause a tree of pathologies. Disease Models & Mechanisms.

[bib24] Dasgupta C, Martinez AM, Zuppan CW, Shah MM, Bailey LL, Fletcher WH (2001). Identification of connexin43 (alpha1) gap junction gene mutations in patients with hypoplastic left heart syndrome by denaturing gradient gel electrophoresis (DGGE). Mutation Research.

[bib25] Deisenroth C, Zhang Y (2011). The ribosomal protein-Mdm2-p53 pathway and energy metabolism. Genes & Cancer.

[bib26] Del Re DP, Yang Y, Nakano N, Cho J, Zhai P, Yamamoto T, Zhang N, Yabuta N, Nojima H, Pan D, Sadoshima J (2013). Yes-associated protein isoform 1 (Yap1) promotes cardiomyocyte survival and growth to protect against myocardial ischemic injury. The Journal of Biological Chemistry.

[bib27] Del Re DP (2014). The hippo signaling pathway: implications for heart regeneration and disease. Clinical and Translational Medicine.

[bib28] Diez-Cuñado M, Wei K, Bushway PJ, Maurya MR, Perera R, Subramaniam S, Ruiz-Lozano P, Mercola M (2018). miRNAs that induce human cardiomyocyte proliferation converge on the hippo pathway. Cell Reports.

[bib29] Elliott DA, Kirk EP, Yeoh T, Chandar S, McKenzie F, Taylor P, Grossfeld P, Fatkin D, Jones O, Hayes P, Feneley M, Harvey RP (2003). Cardiac homeobox gene NKX2-5 mutations and congenital heart disease: associations with atrial septal defect and hypoplastic left heart syndrome. Journal of the American College of Cardiology.

[bib30] Evans SM, Yelon D, Conlon FL, Kirby ML (2010). Myocardial lineage development. Circulation Research.

[bib31] Ewels PA, Peltzer A, Fillinger S, Patel H, Alneberg J, Wilm A, Garcia MU, Di Tommaso P, Nahnsen S (2020). The nf-core framework for community-curated bioinformatics pipelines. Nature Biotechnology.

[bib32] Fink M, Callol-Massot C, Chu A, Ruiz-Lozano P, Izpisua Belmonte JC, Giles W, Bodmer R, Ocorr K (2009). A new method for detection and quantification of heartbeat parameters in *Drosophila*, zebrafish, and embryonic mouse hearts. BioTechniques.

[bib33] Fischer M (2017). Census and evaluation of p53 target genes. Oncogene.

[bib34] Flucke U, van Noesel MM, Siozopoulou V, Creytens D, Tops BBJ, van Gorp JM, Hiemcke-Jiwa LS (2021). *EWSR1*-The most common rearranged gene in soft tissue lesions, which also occurs in different bone lesions: an updated review. Diagnostics.

[bib35] Furth N, Aylon Y, Oren M (2018). p53 shades of Hippo. Cell Death and Differentiation.

[bib36] Gaber N, Gagliardi M, Patel P, Kinnear C, Zhang C, Chitayat D, Shannon P, Jaeggi E, Tabori U, Keller G, Mital S (2013). Fetal reprogramming and senescence in hypoplastic left heart syndrome and in human pluripotent stem cells during cardiac differentiation. The American Journal of Pathology.

[bib37] Gagnon JA, Valen E, Thyme SB, Huang P, Akhmetova L, Pauli A, Montague TG, Zimmerman S, Richter C, Schier AF (2014). Efficient mutagenesis by Cas9 protein-mediated oligonucleotide insertion and large-scale assessment of single-guide RNAs. PLOS ONE.

[bib38] Gifford CA, Ranade SS, Samarakoon R, Salunga HT, de Soysa TY, Huang Y, Zhou P, Elfenbein A, Wyman SK, Bui YK, Cordes Metzler KR, Ursell P, Ivey KN, Srivastava D (2019). Oligogenic inheritance of a human heart disease involving a genetic modifier. Science.

[bib39] Gordon BM, Rodriguez S, Lee M, Chang RK (2008). Decreasing number of deaths of infants with hypoplastic left heart syndrome. The Journal of Pediatrics.

[bib40] Grodecká L, Kramárek M, Lockerová P, Kováčová T, Ravčuková B, Richterová R, Kyselová K, Augste E, Freiberger T (2014). No major effect of the CDH1 c.2440‐6C>G mutation on splicing detected in last exon‐specific splicing minigene assay. Genes, Chromosomes & Cancer.

[bib41] Grossfeld P, Ye M, Harvey R (2009). Hypoplastic left heart syndrome: new genetic insights. Journal of the American College of Cardiology.

[bib42] Hallier B, Hoffmann J, Roeder T, Tögel M, Meyer H, Paululat A (2015). The bHLH transcription factor hand regulates the expression of genes critical to heart and muscle function in *Drosophila melanogaster*. PLOS ONE.

[bib43] Han Z, Yi P, Li X, Olson EN (2006). Hand, an evolutionarily conserved bHLH transcription factor required for *Drosophila* cardiogenesis and hematopoiesis. Development.

[bib44] Hoshijima K, Jurynec MJ, Klatt Shaw D, Jacobi AM, Behlke MA, Grunwald DJ (2019). Highly efficient CRISPR-Cas9-based methods for generating deletion mutations and F0 embryos that lack gene function in zebrafish. Developmental Cell.

[bib45] Hu Y, Flockhart I, Vinayagam A, Bergwitz C, Berger B, Perrimon N, Mohr SE (2011). An integrative approach to ortholog prediction for disease-focused and other functional studies. BMC Bioinformatics.

[bib46] Huang CJ, Tu CT, Hsiao CD, Hsieh FJ, Tsai HJ (2003). Germ-line transmission of a myocardium-specific GFP transgene reveals critical regulatory elements in the cardiac myosin light chain 2 promoter of zebrafish. Developmental Dynamics.

[bib47] Huang G-Y, Xie L-J, Linask KL, Zhang C, Zhao X-Q, Yang Y, Zhou G-M, Wu Y-J, Marquez-Rosado L, McElhinney DB, Goldmuntz E, Liu C, Lampe PD, Chatterjee B, Lo CW (2011). Evaluating the role of connexin43 in congenital heart disease: Screening for mutations in patients with outflow tract anomalies and the analysis of knock-in mouse models. Journal of Cardiovascular Disease Research.

[bib48] Ikeda F, Yoshida K, Toki T, Uechi T, Ishida S, Nakajima Y, Sasahara Y, Okuno Y, Kanezaki R, Terui K, Kamio T, Kobayashi A, Fujita T, Sato-Otsubo A, Shiraishi Y, Tanaka H, Chiba K, Muramatsu H, Kanno H, Ohga S, Ohara A, Kojima S, Kenmochi N, Miyano S, Ogawa S, Ito E (2017). Exome sequencing identified *RPS15A* as a novel causative gene for Diamond-Blackfan anemia. Haematologica.

[bib49] Irion U, Krauss J, Nüsslein-Volhard C (2014). Precise and efficient genome editing in zebrafish using the CRISPR/Cas9 system. Development.

[bib50] Jin SC, Homsy J, Zaidi S, Lu Q, Morton S, DePalma SR, Zeng X, Qi H, Chang W, Sierant MC, Hung WC, Haider S, Zhang J, Knight J, Bjornson RD, Castaldi C, Tikhonoa IR, Bilguvar K, Mane SM, Sanders SJ, Mital S, Russell MW, Gaynor JW, Deanfield J, Giardini A, Porter GA, Srivastava D, Lo CW, Shen Y, Watkins WS, Yandell M, Yost HJ, Tristani-Firouzi M, Newburger JW, Roberts AE, Kim R, Zhao H, Kaltman JR, Goldmuntz E, Chung WK, Seidman JG, Gelb BD, Seidman CE, Lifton RP, Brueckner M (2017). Contribution of rare inherited and de novo variants in 2,871 congenital heart disease probands. Nature Genetics.

[bib51] Kang J, Brajanovski N, Chan KT, Xuan J, Pearson RB, Sanij E (2021). Ribosomal proteins and human diseases: molecular mechanisms and targeted therapy. Signal Transduction and Targeted Therapy.

[bib52] Karczewski KJ, Francioli LC, Tiao G, Cummings BB, Alföldi J, Wang Q, Collins RL, Laricchia KM, Ganna A, Birnbaum DP, Gauthier LD, Brand H, Solomonson M, Watts NA, Rhodes D, Singer-Berk M, England EM, Seaby EG, Kosmicki JA, Walters RK, Tashman K, Farjoun Y, Banks E, Poterba T, Wang A, Seed C, Whiffin N, Chong JX, Samocha KE, Pierce-Hoffman E, Zappala Z, O’Donnell-Luria AH, Minikel EV, Weisburd B, Lek M, Ware JS, Vittal C, Armean IM, Bergelson L, Cibulskis K, Connolly KM, Covarrubias M, Donnelly S, Ferriera S, Gabriel S, Gentry J, Gupta N, Jeandet T, Kaplan D, Llanwarne C, Munshi R, Novod S, Petrillo N, Roazen D, Ruano-Rubio V, Saltzman A, Schleicher M, Soto J, Tibbetts K, Tolonen C, Wade G, Talkowski ME, Aguilar Salinas CA, Ahmad T, Albert CM, Ardissino D, Atzmon G, Barnard J, Beaugerie L, Benjamin EJ, Boehnke M, Bonnycastle LL, Bottinger EP, Bowden DW, Bown MJ, Chambers JC, Chan JC, Chasman D, Cho J, Chung MK, Cohen B, Correa A, Dabelea D, Daly MJ, Darbar D, Duggirala R, Dupuis J, Ellinor PT, Elosua R, Erdmann J, Esko T, Färkkilä M, Florez J, Franke A, Getz G, Glaser B, Glatt SJ, Goldstein D, Gonzalez C, Groop L, Haiman C, Hanis C, Harms M, Hiltunen M, Holi MM, Hultman CM, Kallela M, Kaprio J, Kathiresan S, Kim BJ, Kim YJ, Kirov G, Kooner J, Koskinen S, Krumholz HM, Kugathasan S, Kwak SH, Laakso M, Lehtimäki T, Loos RJF, Lubitz SA, Ma RCW, MacArthur DG, Marrugat J, Mattila KM, McCarroll S, McCarthy MI, McGovern D, McPherson R, Meigs JB, Melander O, Metspalu A, Neale BM, Nilsson PM, O’Donovan MC, Ongur D, Orozco L, Owen MJ, Palmer CNA, Palotie A, Park KS, Pato C, Pulver AE, Rahman N, Remes AM, Rioux JD, Ripatti S, Roden DM, Saleheen D, Salomaa V, Samani NJ, Scharf J, Schunkert H, Shoemaker MB, Sklar P, Soininen H, Sokol H, Spector T, Sullivan PF, Suvisaari J, Tai ES, Teo YY, Tiinamaija T, Tsuang M, Turner D, Tusie-Luna T, Vartiainen E, Vawter MP, Ware JS, Watkins H, Weersma RK, Wessman M, Wilson JG, Xavier RJ, Neale BM, Daly MJ, MacArthur DG, Genome Aggregation Database Consortium (2020). The mutational constraint spectrum quantified from variation in 141,456 humans. Nature.

[bib53] Kathiriya IS, Rao KS, Iacono G, Devine WP, Blair AP, Hota SK, Lai MH, Garay BI, Thomas R, Gong HZ, Wasson LK, Goyal P, Sukonnik T, Hu KM, Akgun GA, Bernard LD, Akerberg BN, Gu F, Li K, Speir ML, Haeussler M, Pu WT, Stuart JM, Seidman CE, Seidman JG, Heyn H, Bruneau BG (2021). Modeling human TBX5 haploinsufficiency predicts regulatory networks for congenital heart disease. Developmental Cell.

[bib54] Konno Y, Toki T, Tandai S, Xu G, Wang R, Terui K, Ohga S, Hara T, Hama A, Kojima S, Hasegawa D, Kosaka Y, Yanagisawa R, Koike K, Kanai R, Imai T, Hongo T, Park MJ, Sugita K, Ito E (2010). Mutations in the ribosomal protein genes in Japanese patients with Diamond-Blackfan anemia. Haematologica.

[bib55] Lee KH, Xu Q, Breitbart RE (1996). A new tinman-related gene, nkx2.7, anticipates the expression of nkx2.5 and nkx2.3 in zebrafish heart and pharyngeal endoderm. Developmental Biology.

[bib56] Liu J, Stainier DYR (2012). Zebrafish in the study of early cardiac development. Circulation Research.

[bib57] Liu Y, Deisenroth C, Zhang Y (2016). RP-MDM2-p53 pathway: linking ribosomal biogenesis and tumor surveillance. Trends in Cancer.

[bib58] Liu X, Yagi H, Saeed S, Bais AS, Gabriel GC, Chen Z, Peterson KA, Li Y, Schwartz MC, Reynolds WT, Saydmohammed M, Gibbs B, Wu Y, Devine W, Chatterjee B, Klena NT, Kostka D, de Mesy Bentley KL, Ganapathiraju MK, Dexheimer P, Leatherbury L, Khalifa O, Bhagat A, Zahid M, Pu W, Watkins S, Grossfeld P, Murray SA, Porter GA, Tsang M, Martin LJ, Benson DW, Aronow BJ, Lo CW (2017). The complex genetics of hypoplastic left heart syndrome. Nature Genetics.

[bib59] Love MI, Huber W, Anders S (2014). Moderated estimation of fold change and dispersion for RNA-seq data with DESeq2. Genome Biology.

[bib60] Mak TW, Hauck L, Grothe D, Billia F (2017). p53 regulates the cardiac transcriptome. PNAS.

[bib61] Martin LJ, Pilipenko V, Benson DW (2018). Role of segregation for variant discovery in multiplex families ascertained by probands with left sided cardiovascular malformations. Frontiers in Genetics.

[bib62] McGowan KA, Pang WW, Bhardwaj R, Perez MG, Pluvinage JV, Glader BE, Malek R, Mendrysa SM, Weissman IL, Park CY, Barsh GS (2011). Reduced ribosomal protein gene dosage and p53 activation in low-risk myelodysplastic syndrome. Blood.

[bib63] Men H, Cai H, Cheng Q, Zhou W, Wang X, Huang S, Zheng Y, Cai L (2021). The regulatory roles of p53 in cardiovascular health and disease. Cellular and Molecular Life Sciences.

[bib64] Mi H, Muruganujan A, Ebert D, Huang X, Thomas PD (2019). PANTHER version 14: more genomes, a new PANTHER GO-slim and improvements in enrichment analysis tools. Nucleic Acids Research.

[bib65] Miao Y, Tian L, Martin M, Paige SL, Galdos FX, Li J, Klein A, Zhang H, Ma N, Wei Y, Stewart M, Lee S, Moonen JR, Zhang B, Grossfeld P, Mital S, Chitayat D, Wu JC, Rabinovitch M, Nelson TJ, Nie S, Wu SM, Gu M (2020). Intrinsic endocardial defects contribute to hypoplastic left heart syndrome. Cell Stem Cell.

[bib66] Miura GI, Yelon D (2011). A guide to analysis of cardiac phenotypes in the zebrafish embryo. Methods in Cell Biology.

[bib67] Monroe TO, Hill MC, Morikawa Y, Leach JP, Heallen T, Cao S, Krijger PHL, de Laat W, Wehrens XHT, Rodney GG, Martin JF (2019). YAP partially reprograms chromatin accessibility to directly induce adult cardiogenesis in vivo. Developmental Cell.

[bib68] Murphy SA, Miyamoto M, Kervadec A, Kannan S, Tampakakis E, Kambhampati S, Lin BL, Paek S, Andersen P, Lee D-I, Zhu R, An SS, Kass DA, Uosaki H, Colas AR, Kwon C (2021). PGC1/PPAR drive cardiomyocyte maturation at single cell level via YAP1 and SF3B2. Nature Communications.

[bib69] Ocorr K, Fink M, Cammarato A, Bernstein SI, Bodmer R (2009). Semi-automated optical heartbeat analysis of small hearts. Journal of Visualized Experiments.

[bib70] Ocorr K, Vogler G, Bodmer R (2014). Methods to assess *Drosophila* heart development, function and aging. Methods.

[bib71] Ollmann M, Young LM, Di Como CJ, Karim F, Belvin M, Robertson S, Whittaker K, Demsky M, Fisher WW, Buchman A, Duyk G, Friedman L, Prives C, Kopczynski C (2000). *Drosophila* p53 is a structural and functional homolog of the tumor suppressor p53. Cell.

[bib72] Paige SL, Galdos FX, Lee S, Chin ET, Ranjbarvaziri S, Feyen DAM, Darsha AK, Xu S, Ryan JA, Beck AL, Qureshi MY, Miao Y, Gu M, Bernstein D, Nelson TJ, Mercola M, Rabinovitch M, Ashley EA, Parikh VN, Wu SM (2020). Patient-specific induced pluripotent stem cells implicate intrinsic impaired contractility in hypoplastic left heart syndrome. Circulation.

[bib73] Pei F, Jiang J, Bai S, Cao H, Tian L, Zhao Y, Yang C, Dong H, Ma Y (2017). Chemical-defined and albumin-free generation of human atrial and ventricular myocytes from human pluripotent stem cells. Stem Cell Research.

[bib74] Pradhan A, Zeng XXI, Sidhwani P, Marques SR, George V, Targoff KL, Chi NC, Yelon D (2017). FGF signaling enforces cardiac chamber identity in the developing ventricle. Development.

[bib75] Qian L, Wythe JD, Liu J, Cartry J, Vogler G, Mohapatra B, Otway RT, Huang Y, King IN, Maillet M, Zheng Y, Crawley T, Taghli-Lamallem O, Semsarian C, Dunwoodie S, Winlaw D, Harvey RP, Fatkin D, Towbin JA, Molkentin JD, Srivastava D, Ocorr K, Bruneau BG, Bodmer R (2011). Tinman/Nkx2-5 acts via miR-1 and upstream of Cdc42 to regulate heart function across species. The Journal of Cell Biology.

[bib76] Ragni CV, Diguet N, Le Garrec JF, Novotova M, Resende TP, Pop S, Charon N, Guillemot L, Kitasato L, Badouel C, Dufour A, Olivo-Marin JC, Trouvé A, McNeill H, Meilhac SM (2017). Amotl1 mediates sequestration of the Hippo effector Yap1 downstream of Fat4 to restrict heart growth. Nature Communications.

[bib77] Raj N, Bam R (2019). Reciprocal crosstalk between YAP1/Hippo pathway and the p53 family proteins: mechanisms and outcomes in cancer. Frontiers in Cell and Developmental Biology.

[bib78] Raudvere U, Kolberg L, Kuzmin I, Arak T, Adler P, Peterson H, Vilo J (2019). g:Profiler: a web server for functional enrichment analysis and conversions of gene lists (2019 update). Nucleic Acids Research.

[bib79] Reller MD, Strickland MJ, Riehle-Colarusso T, Mahle WT, Correa A (2008). Prevalence of congenital heart defects in metropolitan Atlanta, 1998-2005. The Journal of Pediatrics.

[bib80] Robu ME, Larson JD, Nasevicius A, Beiraghi S, Brenner C, Farber SA, Ekker SC (2007). p53 activation by knockdown technologies. PLOS Genetics.

[bib81] Schroeder AM, Allahyari M, Vogler G, Missinato MA, Nielsen T, Yu MS, Theis JL, Larsen LA, Goyal P, Rosenfeld JA, Nelson TJ, Olson TM, Colas AR, Grossfeld P, Bodmer R (2019). Model system identification of novel congenital heart disease gene candidates: focus on RPL13. Human Molecular Genetics.

[bib82] Schumacher JA, Bloomekatz J, Garavito-Aguilar ZV, Yelon D (2013). tal1 Regulates the formation of intercellular junctions and the maintenance of identity in the endocardium. Developmental Biology.

[bib83] Szklarczyk D, Gable AL, Lyon D, Junge A, Wyder S, Huerta-Cepas J, Simonovic M, Doncheva NT, Morris JH, Bork P, Jensen LJ, von Mering C (2019). STRING v11: protein-protein association networks with increased coverage, supporting functional discovery in genome-wide experimental datasets. Nucleic Acids Research.

[bib84] Talbot JC, Amacher SL (2014). A streamlined CRISPR pipeline to reliably generate zebrafish frameshifting alleles. Zebrafish.

[bib85] Tanaka M, Yamaguchi S, Yamazaki Y, Kinoshita H, Kuwahara K, Nakao K, Jay PY, Noda T, Nakamura T (2015). Somatic chromosomal translocation between Ewsr1 and Fli1 loci leads to dilated cardiomyopathy in a mouse model. Scientific Reports.

[bib86] Targoff KL, Schell T, Yelon D (2008). Nkx genes regulate heart tube extension and exert differential effects on ventricular and atrial cell number. Developmental Biology.

[bib87] Theis JL, Hrstka SCL, Evans JM, O’Byrne MM, de Andrade M, O’Leary PW, Nelson TJ, Olson TM (2015a). Compound heterozygous NOTCH1 mutations underlie impaired cardiogenesis in a patient with hypoplastic left heart syndrome. Human Genetics.

[bib88] Theis JL, Zimmermann MT, Evans JM, Eckloff BW, Wieben ED, Qureshi MY, O’Leary PW, Olson TM (2015b). Recessive MYH6 mutations in hypoplastic left heart with reduced ejection fraction. Circulation. Cardiovascular Genetics.

[bib89] Theis JL, Vogler G, Missinato MA, Li X, Nielsen T, Zeng X-XI, Martinez-Fernandez A, Walls SM, Kervadec A, Kezos JN, Birker K, Evans JM, O’Byrne MM, Fogarty ZC, Terzic A, Grossfeld P, Ocorr K, Nelson TJ, Olson TM, Colas AR, Bodmer R (2020). Patient-specific genomics and cross-species functional analysis implicate LRP2 in hypoplastic left heart syndrome. eLife.

[bib90] Tiu GC, Kerr CH, Forester CM, Krishnarao PS, Rosenblatt HD, Raj N, Lantz TC, Zhulyn O, Bowen ME, Shokat L, Attardi LD, Ruggero D, Barna M (2021). A p53-dependent translational program directs tissue-selective phenotypes in a model of ribosomopathies. Developmental Cell.

[bib91] Tögel M, Meyer H, Lehmacher C, Heinisch JJ, Pass G, Paululat A (2013). The bHLH transcription factor hand is required for proper wing heart formation in Drosophila. Developmental Biology.

[bib92] Uechi T, Nakajima Y, Nakao A, Torihara H, Chakraborty A, Inoue K, Kenmochi N (2006). Ribosomal protein gene knockdown causes developmental defects in zebrafish. PLOS ONE.

[bib93] Vlachos A, Osorio DS, Atsidaftos E, Kang J, Lababidi ML, Seiden HS, Gruber D, Glader BE, Onel K, Farrar JE, Bodine DM, Aspesi A, Dianzani I, Ramenghi U, Ellis SR, Lipton JM (2018). Increased prevalence of congenital heart disease in children with Diamond Blackfan anemia suggests unrecognized Diamond Blackfan anemia as a cause of congenital heart disease in the general population: A report of the Diamond Blackfan Anemia Registry. Circulation. Genomic and Precision Medicine.

[bib94] Vogler G (2021). Zenodo.

[bib95] Vogler G (2025). Software Heritage.

[bib96] von Gise A, Lin Z, Schlegelmilch K, Honor LB, Pan GM, Buck JN, Ma Q, Ishiwata T, Zhou B, Camargo FD, Pu WT (2012). YAP1, the nuclear target of Hippo signaling, stimulates heart growth through cardiomyocyte proliferation but not hypertrophy. PNAS.

[bib97] Wang J, Liu S, Heallen T, Martin JF (2018). The Hippo pathway in the heart: pivotal roles in development, disease, and regeneration. Nature Reviews Cardiology.

[bib98] Xiao Y, Leach J, Wang J, Martin JF (2016). Hippo/Yap signaling in cardiac development and regeneration. Current Treatment Options in Cardiovascular Medicine.

[bib99] Xu X, Jin K, Bais AS, Zhu W, Yagi H, Feinstein TN, Nguyen PK, Criscione JD, Liu X, Beutner G, Karunakaran KB, Rao KS, He H, Adams P, Kuo CK, Kostka D, Pryhuber GS, Shiva S, Ganapathiraju MK, Porter GA, Lin JHI, Aronow B, Lo CW (2022). Uncompensated mitochondrial oxidative stress underlies heart failure in an iPSC-derived model of congenital heart disease. Cell Stem Cell.

[bib100] Yagi H, Liu X, Gabriel GC, Wu Y, Peterson K, Murray SA, Aronow BJ, Martin LJ, Benson DW, Lo CW (2018). The genetic landscape of hypoplastic left heart syndrome. Pediatric Cardiology.

[bib101] Yaich L, Ooi J, Park M, Borg JP, Landry C, Bodmer R, Margolis B (1998). Functional analysis of the Numb phosphotyrosine-binding domain using site-directed mutagenesis. The Journal of Biological Chemistry.

[bib102] Yang K, Yang J, Yi J (2018). Nucleolar Stress: hallmarks, sensing mechanism and diseases. Cell Stress.

[bib103] Ye M, Coldren C, Liang X, Mattina T, Goldmuntz E, Benson DW, Ivy D, Perryman MB, Garrett-Sinha LA, Grossfeld P (2010). Deletion of ETS-1, a gene in the Jacobsen syndrome critical region, causes ventricular septal defects and abnormal ventricular morphology in mice. Human Molecular Genetics.

[bib104] Yu L, Daniels JP, Wu H, Wolf MJ (2015). Cardiac hypertrophy induced by active Raf depends on Yorkie-mediated transcription. Science Signaling.

[bib105] Yu MS, Spiering S, Colas AR (2018). Generation of first heart field-like cardiac progenitors and ventricular-like cardiomyocytes from human pluripotent stem cells. Journal of Visualized Experiments.

[bib106] Yuan LL, Green A, David L, Dozier C, Récher C, Didier C, Tamburini J, Manenti S (2014). Targeting CHK1 inhibits cell proliferation in FLT3-ITD positive acute myeloid leukemia. Leukemia Research.

[bib107] Zanon A, Kalvakuri S, Rakovic A, Foco L, Guida M, Schwienbacher C, Serafin A, Rudolph F, Trilck M, Grünewald A, Stanslowsky N, Wegner F, Giorgio V, Lavdas AA, Bodmer R, Pramstaller PP, Klein C, Hicks AA, Pichler I, Seibler P (2017). SLP-2 interacts with Parkin in mitochondria and prevents mitochondrial dysfunction in Parkin-deficient human iPSC-derived neurons and *Drosophila*. Human Molecular Genetics.

[bib108] Zeng XXI, Yelon D (2014). Cadm4 restricts the production of cardiac outflow tract progenitor cells. Cell Reports.

[bib109] Zhang Z, Li M, Wang H, Agrawal S, Zhang R (2003). Antisense therapy targeting MDM2 oncogene in prostate cancer: Effects on proliferation, apoptosis, multiple gene expression, and chemotherapy. PNAS.

[bib110] Zhang Y, Lu H (2009). Signaling to p53: ribosomal proteins find their way. Cancer Cell.

[bib111] Zhou X, Liao WJ, Liao JM, Liao P, Lu H (2015). Ribosomal proteins: functions beyond the ribosome. Journal of Molecular Cell Biology.

